# The burden of the digital environment: a systematic review on organization-directed workplace interventions to mitigate physician burnout

**DOI:** 10.1093/jamia/ocaa301

**Published:** 2021-01-19

**Authors:** Kelly J Thomas Craig, Van C Willis, David Gruen, Kyu Rhee, Gretchen P Jackson

**Affiliations:** 1 Center for AI, Research, and Evaluation, IBM Watson Health, Cambridge, Massachusetts, USA; 2 Vanderbilt University Medical Center, Nashville, Tennessee, USA

**Keywords:** burnout, electronic health records, quality improvement, workflow, team-based care

## Abstract

**Objective:**

To conduct a systematic review identifying workplace interventions that mitigate physician burnout related to the digital environment including health information technologies (eg, electronic health records) and decision support systems) with or without the application of advanced analytics for clinical care.

**Materials and Methods:**

Literature published from January 1, 2007 to June 3, 2020 was systematically reviewed from multiple databases and hand searches. Subgroup analysis identified relevant physician burnout studies with interventions examining digital tool burden, related workflow inefficiencies, and measures of burnout, stress, or job satisfaction in all practice settings.

**Results:**

The search strategy identified 4806 citations of which 81 met inclusion criteria. Thirty-eight studies reported interventions to decrease digital tool burden. Sixty-eight percent of these studies reported improvement in burnout and/or its proxy measures. Burnout was decreased by interventions that optimized technologies (primarily electronic health records), provided training, reduced documentation and task time, expanded the care team, and leveraged quality improvement processes in workflows.

**Discussion:**

The contribution of digital tools to physician burnout can be mitigated by careful examination of usability, introducing technologies to save or optimize time, and applying quality improvement to workflows.

**Conclusion:**

Physician burnout is not reduced by technology implementation but can be mitigated by technology and workflow optimization, training, team expansion, and careful consideration of factors affecting burnout, including specialty, practice setting, regulatory pressures, and how physicians spend their time.

## INTRODUCTION

Clinician burnout was considered a global health crisis before coronavirus disease 2019 (COVID-19) with more than 60% of providers reporting at least 1 symptom of burnout.^1^^,^^2^ However, the pandemic has compounded the emotional, physical, and mental exhaustion for healthcare providers as stressors within the healthcare system have been exacerbated. The mental health consequences, particularly for clinicians on the front line, may be significant. Acute increases in workplace stressors may increase the prevalence of post-traumatic stress disorder (PTSD); notably, the drivers of PTSD and burnout are similar with overlapping harmful impacts on provider health.^3^ In the current setting with COVID-19, resiliency techniques targeting the individual are important, but insufficient to overcome systemic challenges that give rise to burnout. Healthcare organizations, now more than ever, need to prioritize their employees’ health and address the drivers of burnout.

Arguably, the primary drivers of burnout for physicians have been related to electronic health records (EHRs) and overwhelming inefficiencies in clinical practice that significantly and negatively impact workflow and patient care.^4^^,^^5^ Physicians experience high fatigue with short, continuous periods of EHR use, which is also associated with inefficiency of EHR use (ie, more clicks and more time) on subsequent cases.^6^ The association of burnout with EHR design and usability has been identified,^7^ in addition to the clerical burden of technology on workload (both cognitive and physical) and its associated workflows.^8^^,^^9^ Despite the general acknowledgement that the evolving digital environment using health information technologies (eg, patient portals, clinical notes, computerized order-entry, electronic prescribing) for both regulatory and administrative purposes has altered physicians’ practice, interventions to lessen associated burnout have not been well characterized. To date, there is a gap in understanding whether physician burnout is improved by interventions designed to decrease the burden of their digital environment and improve related clinical workflow efficiency.

We conducted a systematic review in 2018 to identify workplace interventions to alleviate burnout;^10^ however given the COVID-19 climate, reducing burnout remains a high priority to safeguard healthcare providers and the patients and system they serve. The scope of published burnout-related interventions continues to exponentially grow, but the connection to the digital environment has not been synthesized. The objective of this study is to perform an update of our systematic review and conduct a subgroup analysis to identify interventions to mitigate digital tool burden and/or its related workflow inefficiencies.

## MATERIALS AND METHODS

**Figure 1.**
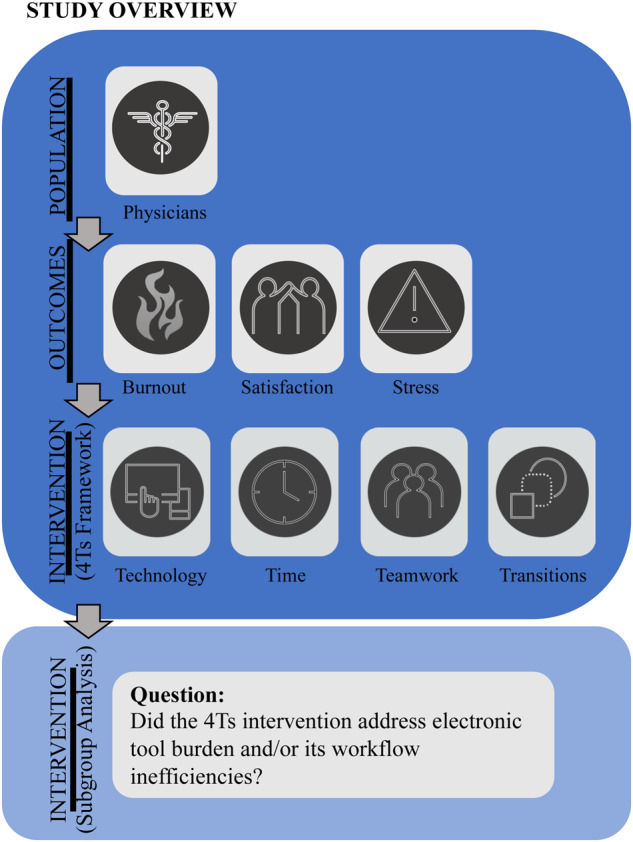
Study overview, description of 4Ts framework, and subgroup analysis.

This study was conducted in accordance with Preferred Reporting Items for Systematic Reviews and Meta-Analyses (PRISMA)^11^ under an *a priori* protocol. This study is an update and subgroup analysis of our systematic review of workplace interventions to mitigate physician burnout.^10^ The objective of the subgroup analysis was to identify and summarize interventions used to address the burden of digital tools and their impact on workflow inefficiencies, whereas the original study results sought to identify *any* type of organization-directed workplace intervention to address burnout (Figure 1).

### Search strategy

Literature was searched from multiple databases (MEDLINE, Embase, and Cochrane Library) on October 3, 2018; an updated search was performed on June 3, 2020 for relevant articles; and the Association for Computing Machinery (ACM) Digital Library was queried on August 17, 2020. Queries were designed to identify physician-specific burnout or burnout proxy (satisfaction and/or stress) outcomes following work, workplace, or workflow interventions. Search limits were set regarding time (2007–2020), English-language, and abstract availability. Within the protocol, the introduction of the Health Information Technology for Economic and Clinical Health (HiTECH) Act of 2009 was a distinguishing time point. Based upon the widespread implementation of HIT post-2009, evidence collected before this date was not of interest based on the assumption that burnout has increased dramatically as a result of organizational changes to accommodate the meaningful use of HIT in clinical care. Hand searches were also conducted to identify literature from key conferences, organization websites, and bibliographies of included studies. Details are provided in Supplementary Tables 1–10.

### Screening process

One investigator (KJTC or VCW) screened all titles and abstracts for eligibility against *a priori* established inclusion criteria (Supplementary Table 11). Included studies examined physician-specific burnout or burnout proxy outcomes (satisfaction and/or stress) following an organization-directed intervention (ie, not individual interventions conducted outside of the workplace, sometimes referred to as physician-directed) whereby comparisons were provided to examine the effect of the intervention in all settings. Studies did not have to be designed to measure the effect of interventions on burnout or its proxy measures but had to capture burnout or burnout-related outcomes as a result of a workplace modification. Studies marked for inclusion were dually screened at the full-text level by 2 independent investigators (KJTC, VCW); any disagreements were resolved by adjudication, or a third reviewer (GPJ) (decision matrix provided in Supplementary Table 12). Additional inclusion criteria were applied to identify studies eligible for the subgroup analysis. Studies were eligible if the intervention specifically considered time management, leveraged team-based care, reexamined key transitions in care processes, and/or primarily, if health information technology (HIT) was adopted, implemented, or optimized with the intention to ease digital burden and/or its related workflow issues. All results were tracked in DistillerSR (Evidence Partners) and EndNote (Clarivate). Interrater reliability was determined by Cohen’s kappa.^12^

### Data extraction and quality assessment

Included studies were extracted (form example in Supplementary Table 13) into *a priori* structured forms by 1 investigator (KJTC) and checked for accuracy and completeness by a second investigator (VCW). Additional data were abstracted for the subgroup analysis to identify study details related to the type of technologies (eg, EHRs, patient portals, decision support systems including any advanced analytics applied) used and associated informatics usability, effectiveness, and impact data, if reported. As with the original review,^10^ studies were categorized into 1 or multiple categories of the 4Ts framework: technology, time, teamwork, and transitions. Technology referred to the implementation or improvement of health information technology, namely EHRs. Time studies involved duty hour restrictions and changes to work schedules or use of time on duty (eg, a program for mindfulness during on-duty time). Teamwork regarded the examination of care team processes and the addition of scribes to the team. Transitions included process improvements and quality initiatives. The Centre for Evidence-Based Medicine derived Oxford Levels of Evidence^13^ were used to assess study quality based on reported study design using their standardized glossary of terms by 2 independent investigators (KJTC, VCW); any disagreements were resolved by adjudication, or a third reviewer (GPJ).

## RESULTS

**Figure 2.**
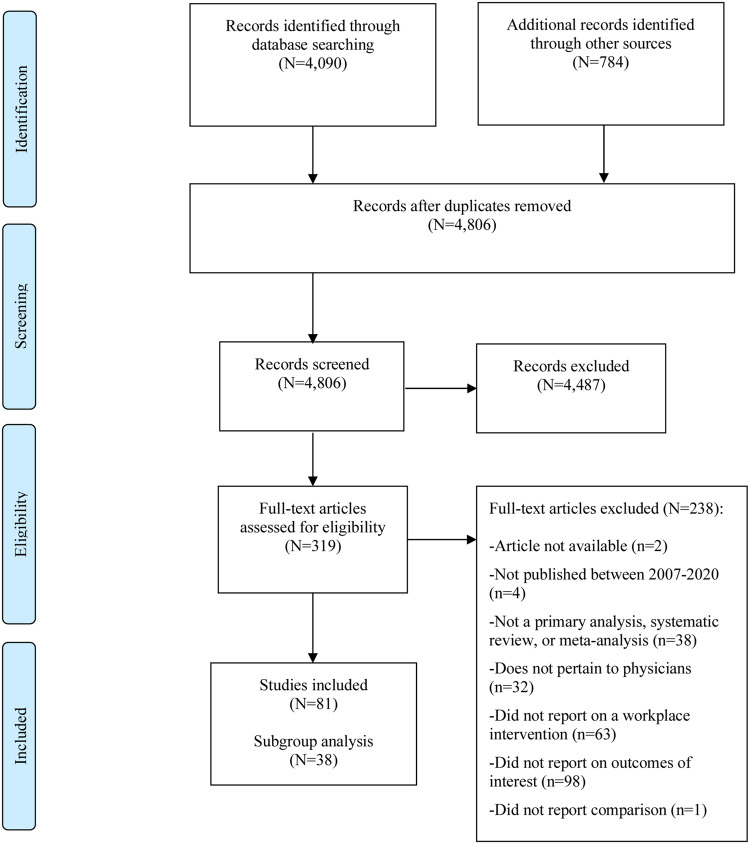
Results of literature search, the Preferred Reporting Items for Systematic Reviews and Meta-Analyses (PRISMA) diagram.^11^

Literature searches yielded 4806 unique citations (Figure 2), of which 319 articles were eligible for full-text screening. Upon full-text screening, 81 articles^10^^,^^14–93^ were included of which 63 were full-length articles and 19 were conference abstracts.^14^^,^^15^^,^^20^^,^^26^^,^^29^^,^^36^^,^^49^^,^^51^^,^^52^^,^^64^^,^^66^^,^^67^^,^^71^^,^^76^^,^^81^^,^^83^^,^^87^^,^^90^^,^^92^ Interrater reliability was 0.76 for full-text screening. As an update to the DeChant et al (2019) study,^10^ 31 additional articles were identified.^10^^,^^18^^,^^19^^,^^21^^,^^23–25^^,^^33^^,^^34^^,^^40–42^^,^^47^^,^^48^^,^^50^^,^^51^^,^^54^^,^^61^^,^^62^^,^^64^^,^^66^^,^^68–70^^,^^72^^,^^75^^,^^80^^,^^81^^,^^85^^,^^87^^,^^89^ Abstractions of included studies can be found in Supplementary Table 14. Thirty-eight studies about digital tool burden and/or related workflow inefficiencies were identified in the subgroup analysis and are the focus of the following synthesis (abstractions in Supplementary Table 15).^10^^,^^14^^,^^17^^,^^20–22^^,^^27–30^^,^^32^^,^^35^^,^^39^^,^^41^^,^^43–46^^,^^49^^,^^51^^,^^53^^,^^56–59^^,^^62–67^^,^^70^^,^^75–77^^,^^83^^,^^92^^,^^93^

### Study and physician characteristics

Characteristics of the 38 studies included in the subgroup analysis are provided in Supplementary Table 15. Most studies (35 studies) were conducted in the US, 1 in the United Kingdom,^17^ and 2 were multinational as systematic reviews.^10^^,^^43^ Study designs included cross-sectional studies (4 studies),^27^^,^^65^^,^^92^^,^^93^ pre-post-intervention surveys (21 studies),^17^^,^^22^^,^^28–30^^,^^35^^,^^44–46^^,^^49^^,^^51^^,^^56^^,^^57^^,^^63^^,^^64^^,^^66^^,^^67^^,^^76^^,^^77^^,^^83^ prospective studies (5 studies),^20^^,^^32^^,^^41^^,^^62^^,^^70^ randomized controlled trials (RCTs) (4 studies),^39^^,^^58^^,^^59^^,^^75^ 2 systematic reviews^10^^,^^43^ and other designs (2 studies).^14^^,^^53^ Comparators were a condition of study inclusion, and the majority of studies provided baseline or preintervention data. Other notable comparator groups included use of standard (eg, nonenhanced) technologies,^20^^,^^48^^,^^62^^,^^75^ paper charting,^66^ no intervention,^58^^,^^59^ and crossover periods without intervention.^39^^,^^70^ Interventions were conducted in multiple settings, but most evaluated primary care (22 studies) physicians and residents.^20^^,^^22^^,^^27–30^^,^^32^^,^^39^^,^^41^^,^^44–46^^,^^56^^,^^58^^,^^59^^,^^64^^,^^65^^,^^70^^,^^77^^,^^83^^,^^92^^,^^93^ Some studies examined specialists (7 studies),^14^^,^^35^^,^^53^^,^^57^^,^^63^^,^^66^^,^^75^ a mix of primary care physicians and specialists (4 studies),^10^^,^^21^^,^^62^^,^^76^ and groups of physicians with unspecified specialties (5 studies).^17^^,^^43^^,^^49^^,^^51^^,^^67^

### Measures of burnout

The gold standard for identifying burnout, the Maslach Burnout Inventory (MBI),^94^ which includes subscales related to depersonalization, emotional exhaustion, and personal accomplishment, was infrequently used (4 studies).^28^^,^^32^^,^^45^^,^^92^ Proxy measures of burnout, including satisfaction and stress, were also abstracted. Studies of satisfaction measured outcomes such as satisfaction, professional fulfillment, well-being, and joy of practice. Studies of stress included outcomes such as stress, psychological (including cognitive) strain, and job distress. Four studies did not report the instrument used to measure burnout-related outcomes.^14^^,^^20^^,^^67^^,^^76^ The majority (17 studies) of authors developed their own surveys,^17^^,^^21^^,^^22^^,^^27^^,^^29^^,^^35^^,^^39^^,^^46^^,^^53^^,^^56^^,^^57^^,^^63–66^^,^^77^^,^^93^ and 2 studies provided only qualitative findings.^49^^,^^83^ As systematic reviews, 2 studies had multiple types of burnout measures.^10^^,^^43^

### Characteristics of organization-directed interventions

**Figure 3.**
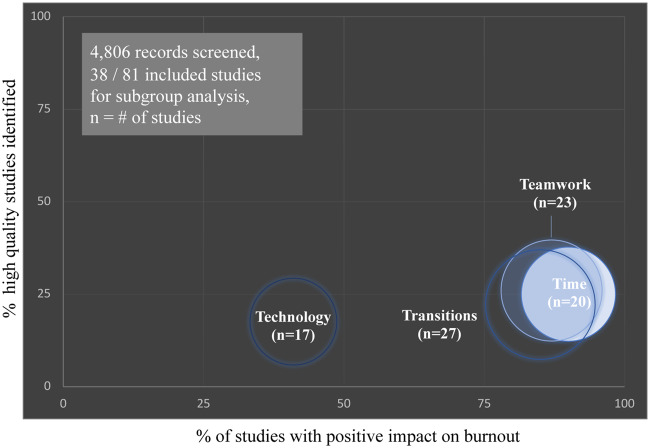
Proportions of interventions with a positive impact on burnout, stratified by intervention type and quality of evidence.

Based on the original review,^10^ interventions were stratified into teamwork, transitions, time, and/or technology (Table 1). Most interventions were assigned to multiple categories with the exception of 10 studies of exclusively technology-centric interventions.^14^^,^^20^^,^^22^^,^^35^^,^^44^^,^^65–67^^,^^75^^,^^93^ Twenty-six (68%) of the 38 studies provided evidence that burnout and its proxy measures of stress and/or satisfaction were improved by a workplace intervention to address workflow inefficiencies in clinical teams that use a digital environment (Table 2).^10^^,^^17^^,^^21^^,^^27–29^^,^^32^^,^^39^^,^^41^^,^^43^^,^^45^^,^^46^^,^^49^^,^^51^^,^^53^^,^^56–59^^,^^63–65^^,^^67^^,^^70^^,^^76^^,^^92^ The scope of this work identified the digital environment including data entry and communication using EHRs (both standard issue and customized), patient portals, disease-management software, clinical decision support, physician order entry, EHR-integrated paging, and clinical task-management systems. The impact on burnout was similar among time, teamwork, and transitions interventions (Figure 3, range 85%–90% of studies with positive outcomes), and these types of interventions were commonly combined. Technology interventions were least effective with 41% of studies reporting improvement in burnout or its proxy measures (Figure 3).

**Table 1.** Study characteristics by intervention type

		Intervention Type(s)								
		**Time** ^a^		**Teamwork** ^b^		**Transitions** ^c^		**Technology** ^d^		
NO.	Reference	Scheduling	Efficiency & productivity	Team-based care	HIT documentation	Process improvement	Workflow change	Adoption	Implementation	Optimization
1	Agha, 2010^14^									
2	Amis, 2018^17^									
3	Babbott, 2013^20^									
4	Baccei, 2020^21^									
5	Beam, 2017^22^									
6	Chapman, 2017^27^									
7	Contratto, 2015^29^									
8	Contratto, 2017^28^									
9	Danila, 2018^30^									
10	DeChant, 2019^10^									
11	Dunn, 2007^32^									
12	Ehrlich, 2016^35^									
13	Gidwani, 2017^39^									
14	Goyal, 2018^41^									
15	Heaton, 2016^43^									
16	Heyworth, 2012^44^									
17	Hung, 2018^45^									
18	Imdieke, 2017^46^									
19	Joseph, 2017^49^									
20	Keefer, 2018^51^									
21	Koshy, 2010^53^									
22	Lapointe, 2018^56^									
23	Lee, 2018^57^									
24	Linzer, 2015^59^									
25	Linzer, 2017^58^									
26	Mazur, 2019^62^									
27	McCormick, 2018^63^									
28	Mehta, 2018^64^									
29	Menachemi, 2009^65^									
30	Michelotti, 2013^66^									
31	Milenkiewicz, 2017^67^									
32	Mishra, 2018^70^									
33	Payne, 2018^75^									
34	Pierce, 2017^76^									
35	Pozdynakova, 2018^77^									
36	Shaw, 2017^83^									
37	Willard-Grace, 2017^92^									
38	Wylie, 2014^93^									
COUNTS		6	17	23	16	10	25	5	11	6

*Note:* Cell shading indicates if intervention contained 1 or more of the following 4Ts intervention categorizations:

a Time: studies involved duty hour restrictions and changes to work schedules or use of time on duty (eg, a program for mindfulness during on-duty time). Scheduling included schedule modifications and flexibility; efficiency & productivity included productivity related interventions regarding efficiency, documentation time, task completion, work interruptions, or patient contact time.

b Teamwork: studies examined care team processes and the addition of scribes to the team. Team-based care included any additional member of the care team including nurses, medical assistants/scribes, other physicians; HIT documentation was limited to studies that used a member of the care team to shift data entry away from the physician.

c Transitions: studies of process improvements and quality initiatives. Process improvement included explicit use of lean methodologies or quality improvement initiatives; workflow change included any intervention that demonstrated a process change whereby a workflow was modified for improvement.

d Technology: studies involved implementation or improvement of health information technology, namely electronic health records. Health information technology included adoption (ie, the sociological process of uptake in usage of a new technology), implementation (ie, installation of new technology and training to use it effectively), or optimization (ie, iterations or changes to technology for improvement).

*Abbreviation:* HIT, health information technology.

**Table 2.** Study results stratified by outcome^a^

**Author, Year** ^b^	Study Design (Oxford Levels of Evidence)	Sample Size (n)	Population	Intervention (4Ts)	Outcomes		
					**Burnout** ^c^	**Satisfaction** ^d^	**Stress** ^e^
Agha, 2010^14^	NR (IV)	9	Specialists^f^^,g,h^	Technology	–	↓*	–
Amis, 2018^17^	Pre-post survey (IV)	13	First year residents	Time, Transitions	–	↑*	–
Babbott, 2013^20^	Prospective (IV)	422	IM and FM physicians	Technology	NS	NS	↑+
Baccei, 2020^21^	Pre-post survey (IV)	6	Radiologists	Time, Teamwork, Transitions	**-**	↑*	**-**
Beam, 2017^22^	Pre-post survey (IV)	158	Physicians	Technology	–	NS	–
Chapman, 2017^27^	Cross-sectional (IV)	886	PC physicians	Teamwork, Transitions	–	↑*	–
Contratto, 2015^29^	Pre-post survey (IV)	9	PC physicians	Time, Teamwork, Transitions	↓*	–	–
Contratto, 2017^28^	Quasi-experimental mixed methods (IV)	7	IM physicians	Time, Teamwork, Transitions	↓*	–	–
Danila, 2018^30^	Pre-post survey (IV)	6	Specialists^g, h^	Teamwork, Transitions	–	NS	–
Dunn, 2007^32^	Prospective study (IV)	22–32	Physicians	Time, Teamwork, Transitions	↓++	NS	–
Ehrlich, 2016^35^	Pre-post survey (IV)	25	Ophthalmologists	Time	–	NS	–
Gidwani, 2017^39^	RCT (IB)	4	Physicians	Time, Teamwork, Transitions	–	↑+++	–
Goyal, 2018^41^	Prospective cohort (IIB)	7	IM physicians (including residents)	Time, Teamwork, Transitions, Technology	–	↑+++	–
Heaton, 2016^43^	Systematic review (IIA)	NA	NA	Time, Teamwork	–	↑*	–
Heyworth, 2012^44^	Pre-post survey (IV)	163	PC and specialty	Technology	–	–	↑+
Hung, 2018^45^ l	Pre-post survey (IV)	680	Physicians	Teamwork, Transitions	↑+++	↑+	↑+++
Imdieke, 2017^46^	Quasi-experimental pre-post intervention study (IV)	2	IM physicians	Time, Teamwork, Transitions	–	↑+++	–
Joseph, 2017^49^	Pre-post survey (IV)	NR	Providers	Time, Transitions Technology	–	↑*	–
Keefer, 2018^51^	Pre-post survey (IV)	NR	Physicians	Time, Teamwork, Transitions	**-**	**-**	↓*
Koshy, 2010^53^	Static-group comparison study (IV)	5	Urologists, residents	Teamwork, Transitions	–	↑+++	–
Lapointe, 2018^56^	Pre-post survey (IV)	25	IM residents	Time, Transitions, Technology	–	↑*	↓*
Lee, 2018^57^	Pre-post survey (IV)	15	Neuroradiologists and fellows	Transitions	–	↑+++	↓+++
Linzer, 2015^59^	Cluster RCT (IIB)	135	FM and IM physicians	Teamwork, Transitions	↓+	NS	NS
Linzer, 2017^58^	Cluster RCT (IIB)	165	FM and IM physicians	Teamwork, Transitions	–	↑+++	↓+++
Mazur, 2019^62^	Prospective cohort (IIB)	38	IM, FM, and specialist^i, j, k^ residents and fellows	Time, Transitions, Technology	**-**	NS	**-**
McCormick, 2018^63^	Pre-post survey (IV)	6	Urologists	Time, Teamwork, Transitions	–	↑+	–
Mehta, 2018^64^	Pre-post survey (IV)	NR	Hospitalists	Time, Teamwork, Transitions, Technology	**-**	↑*	**-**
Menachemi, 2009^65^	Cross-sectional (IV)	4,203	PC physicians and clinical specialists	Technology	–	↑++	–
Michelotti, 2013^66^	Pre-post survey (IV)	59	Faculty ophthalmologists	Technology	**-**	↓+++	**-**
Milenkiewicz, 2017^67^	Pre-post survey (IV)	NR	Physicians	Time, Technology	–	↑*	–
Mishra, 2018^70^	Crossover prospective cohort study (IIB)	18	PC providers	Time, Teamwork, Transitions	**-**	↑*	–
Payne, 2018^75^	RCT (IIB)	31	IM residents and attending hospitalists	Technology	**-**	NS	–
Pierce, 2017^76^	Pre-post survey (IV)	55	Physicians and advanced practice clinicians	Teamwork, Transitions	↓*	–	–
Pozdnyakova, 2018^77^	Prospective, pre-post-pilot study (IV)	6	General IM faculty	Teamwork, Transitions, Technology	NS	–	–
Shaw, 2017^83^	Pre-post survey (IV)	NR	Medical doctors	Teamwork, Transitions	–	NS	–
Willard-Grace, 2017^92^	Cross-sectional (IV)	236	Clinicians	Time, Teamwork, Transitions	↓++	–	–
Wylie, 2014^93^	Cross-sectional (IV)	2365	PC providers	Technology	–	↓+	–

*Note:* The arrows indicate the directionality of the intervention on the effect of burnout, satisfaction, and stress. Improvements are denoted by green color where the associations of the intervention on burnout or proxy measures were statistically significant (see below). Red color indicates the intervention did not improve the burnout or proxy measure (interpreted by a nonsignificant result or *P* values were not provided) or resulted in detractions whereby the outcome measure worsened with the intervention. White content with dashes indicates no data was reported. Levels of evidence: IB, Individual randomized controlled trial (RCT) (with narrow confidence interval); IIA, Systematic review with homogeneity; IIB, Individual cohort study (including low RCT; eg, < 80% follow-up); IV, Case-series, poor-quality cohort, case-control studies, and systematic review with heterogeneity.

a Table adapted from DeChant et al (2019).^10^

b DeChant et al. (2019) was not included in this table as the relevant interventions are already included in the table under the original author names.^10^

c Burnout includes overall burnout, emotional exhaustion, depersonalization, personal accomplishment, and cynicism.

d Satisfaction includes outcomes reported as satisfaction, professional fulfillment, well-being, and joy of practice.

e Stress includes outcomes reported as stress, psychological strain, and job distress.

f Pulmonology, ^g^rheumatology, ^h^endocrinology, ^i^pediatrics, ^j^surgery, or ^k^other specialists.

l Hung et al. describe findings of increased engagement, teamwork, and stress suggesting that their work redesign improved physician experiences, but not sufficiently to overcome workflow challenges linked to stress and burnout.

Symbols: + = *P* < .05; ++ = *P* < .01; +++ = *P* < .001; NS = Not significant; * = No *P* value reported. FM = Family medicine; IM = Internal medicine; NA = Not applicable; NR = Not reported; NS = Not significant; PC = Primary care.

### Study quality

The evaluation of study quality using the Oxford Levels of Evidence^13^ is presented in Supplementary Table 15. The majority (30 studies) were categorized as level IV studies, which includes case series, cross-sectional, poor quality cohort, and pre-post-test single arm studies. Higher quality studies were limited to 1 IB (individual RCT with narrow confidence interval),^39^ 1 IIA (systematic review with homogeneity and meta-analysis),^10^^,^^43^ and 6 IIB (individual cohort including low-quality RCTs with less than 80% follow-up, or systematic review with heterogeneity) categorizations.^41^^,^^58^^,^^59^^,^^62^^,^^70^^,^^75^ Overall, the levels of evidence identified indicate a preponderance of low-quality studies based on study design and corresponding low grades (eg, grade of C, as majority were level IV studies) of (perceived) recommendations.

### Expansion of the care team to give time back to physicians

The most frequent interventions (27 studies) were those that regarded process improvements with the explicit use of lean methodologies or quality improvement (QI) initiatives, and/or workflow changes. QI and lean-based interventions were less successful than general workflow changes, as most QI studies had mixed results^32^^,^^45^^,^^59^ or no significant impact.^21^^,^^51^^,^^62^ Three studies demonstrated that process improvements had overall good outcomes.^49^^,^^57^^,^^58^ One high-quality study identified that burnout was more likely to improve when workflow redesign was part of a QI initiative that addressed physicians’ concerns; a significant reduction in burnout was seen following implementation of QIs around routine preventive screening processes and medication reconciliation in primary care settings.^59^ Another QI intervention that examined the workplace environment of radiologists to reduce both workload and frequency of disruptions increased workplace satisfaction and reduced stress.^57^ Lastly, to reboot the “joy in the practice of medicine,” a process improvement intervention to enhance EHR efficiency increased job satisfaction.^49^ Other process improvement interventions were centered around operationalizing the care team that included: colocation of care teams,^45^ consideration of communication practices,^58^^,^^59^ redesign of responsibilities within the care team and its clinical workflows to improve efficiency,^21^^,^^32^^,^^45^^,^^51^^,^^58^^,^^59^ and the evaluation of electronic data entry and its management.^21^^,^^45^^,^^59^^,^^62^

With the exception of 5 studies,^17^^,^^49^^,^^56^^,^^57^^,^^62^ QI processes and workflow modifications were frequently combined with an intervention to expand the care team to primarily add clerical support by medical assistants/scribes (20 studies).^10^^,^^27–30^^,^^39^^,^^41^^,^^43^^,^^45^^,^^46^^,^^51^^,^^53^^,^^58^^,^^59^^,^^63^^,^^64^^,^^70^^,^^77^^,^^83^^,^^92^ The expanded care team, namely scribes, facilitated electronic data entry for documentation during clinical encounters, such as previsit planning, visit notes (such as history, physical examination findings, laboratory, and/or imaging results), assessments and plans, instructions and education for patients, referrals, and nursing orders. Other team-based interventions improved team dynamics^21^^,^^32^^,^^41^^,^^76^ or hired additional full-time faculty to support and supervise residents to ease their workload, reduced attending-to-patient ratios, and increased number of rounding teams.^51^ Generally, these combinatory interventions decreased burnout,^10^^,^^28^^,^^29^^,^^32^^,^^59^^,^^76^^,^^92^ increased job satisfaction,^10^^,^^27^^,^^39^^,^^41^^,^^43^^,^^45^^,^^46^^,^^49^^,^^53^^,^^57^^,^^58^^,^^63^^,^^64^ and/or decreased stress^10^^,^^56–58^ in physicians.

Many interventions had implicit connections to time (20 studies), mostly considering how clerical tasks within the digital environment could be shifted from the physician to the expanded care team. Modified workflows in expanded care teams improved clinical efficiency and/or productivity, and some studies explicitly measured these outcomes by conducting time and motion studies (17 studies, Table 2). Productivity and/or efficiency were generally improved using scribes for data entry,^28^^,^^29^^,^^43^^,^^63^^,^^64^ and physicians’ task completion and/or documentation time during or after clinic hours and on weekends were decreased significantly.^28^^,^^29^^,^^43^^,^^46^^,^^70^ Additionally, the use of scribes closed patient encounters on average 8.9 days sooner.^63^ One study estimated that physician documentation time was reduced by 50% with scribe implementation, which was a time savings that could be reallocated to patient-facing interactions.^46^ Similarly, improved documentation efficiency with scribe implementation provided “enough” face time with patients,^39^ whereby greater than 75% of the clinical encounter was spent interacting with patients.^70^ Time savings were also noted by interventions without the use of scribes; interventions to improve clinician documentation efficiency using standardized templates resulted in a reduction of 1500 keystrokes per day per provider^49^ and decreased total documentation time 18%–35%.^67^ Other successful time interventions without the use of scribes decreased the frequency of work interruptions^41^^,^^56^ (1 translated to 72.5 physician hours regained in a 3-month intervention period),^56^ reduced number of prescribing tasks for residents listed in a computerized hospital management task system,^17^ and improved physician scheduling to convalesce clinical team interactions via enhanced work–life balance and decreased burnout.^10^^,^^32^^,^^92^

### Leveraging technology to support clinical workflows and teams

Health information technology interventions were stratified into implementation (ie, installation of new technology and training provided to use it effectively) (11 studies), adoption (ie, uptake in usage of a new technology) (5 studies), and/or optimization (ie, iterations or changes to technology for improvement) (6 studies) as shown in Table 1. In addition to burnout outcomes, qualitative and quantitative analyses regarding usability metrics of performance and/or satisfaction (including physician, practice, patient, and IT) were captured in 14 of the 17 studies categorized as technology.^14^^,^^20^^,^^22^^,^^35^^,^^41^^,^^44^^,^^49^^,^^56^^,^^62^^,^^65–67^^,^^75^^,^^93^ Technology adoption and implementation studies generally had no effect on or worsened burnout and its proxy measures,^14^^,^^20^^,^^22^^,^^35^^,^^44^^,^^66^^,^^75^^,^^93^ whereas interventions to optimize the use of technology by design improvement and/or provision of user training were generally effective at reducing burnout, albeit some only qualitatively.^10^^,^^49^^,^^56^^,^^64^^,^^67^ Examples of improvements included usability and agile methodologies to standardize documentation processes in workflows.^49^^,^^62^^,^^77^ Tailored interventions to customize EHRs decreased data entry time by limiting keystrokes and/or mouse clicks through iteratively adjusting the tool’s performance based on user experience and feedback to the design team.^64^^,^^67^ Pre-post-qualitative surveys examining usability of technology optimization and subsequent modified digitally related workflows improved personal, professional, or practice satisfaction.^49^^,^^56^^,^^64^^,^^67^ Only 4 studies incorporated advanced analytics in technology interventions and none had statistically significant positive impacts on burnout. Two studies examined speech recognition algorithms for EHR documentation,^49^^,^^75^ and 2 studies examined enhanced EHR usability with the addition of decision support systems.^22^^,^^62^

## DISCUSSION

This study is the first comprehensive systematic review of interventions to address clinician burnout with a focus on technologies and the digital environment. Many single-site studies have documented the effects of digital technologies on clinician burnout, but evidence about the effectiveness of mitigation strategies has not been synthesized. This systematic review and subgroup analysis summarized the evidence about both successful and ineffective strategies for addressing burnout, factors influencing technology-related burnout, and important scientific gaps in the evidence base. Burnout was decreased by interventions that optimized technology (primarily EHRs), reduced documentation and task time, expanded care teams, and leveraged quality improvement processes to enhance workflows during key transitions in patient care.

This systematic review elucidated several factors that significantly influence the effects of the digital environment on physician burnout, including clinical specialty, practice setting, requirements for compliance and reimbursement, and how physicians spend their time. The disproportionate use of digital tools by certain physician specialties and practice settings is one important consideration. Specialties such as cardiology, urology, and family practice have demonstrated higher EHR use as have practices owned by Health Maintenance Organizations.^95^ Implementation of hospital-wide picture archiving and communication systems have shown improvements in information availability and reporting times while simultaneously isolating the radiologists who use them from interaction with consulting physicians.^96^ Moreover, generic, commercial EHRs have inadequately responded to the needs and challenges that clinical specialties require, but stand-alone specialty EHR systems (without interoperability) have posed additional challenges for physicians and healthcare systems. An effective compromise will likely enable specialty-specific customizations while maintaining standards that support interoperability. Adoption of information and computer technologies for Meaningful Use and documentation required for billing may have perceived improvements in healthcare, but not without concomitant challenges including substantial, low-value digital documentation.

The impact of digital technologies on how physicians spend their time has been shown to affect multiple key domains including productivity and revenue,^97^ job and patient satisfaction,^39^ and personal well-being.^98^ This review identified several interventions to decrease digital tool use and potentially redirect time for face-to-face patient contact and improvements in work-life balance; many of those studies performed time and motion analyses to understand the impact of workflow modification on physicians’ time by proxy of measuring duration of digital tool task completion. Scribe implementation decreased physician documentation hours,^28^^,^^29^^,^^39^^,^^43^^,^^46^^,^^63^^,^^64^^,^^70^ including after-hours and weekends; however, schedule modifications were also leveraged in lean methodologies to provide flexibility for physicians to improve their personal well-being and prevent work overload by providing in-house evening and overnight resident supervision, reducing patient-to-attending ratio, and increasing number of rounding teams.^51^

Stressors in the workplace, particularly those related to the use of health information technologies (ie, “technostress”^99^), are major sources of physician dissatisfaction and burnout, but they can often be mitigated by improvements in the quality and timing of training.^100^ Technostress can increase as complex technologies are integrated into team-based workflows, and when compared to paper documentation, there is a steep learning curve for their effective use. A few studies identified in this review have used advanced training as a mechanism to improve the skill discrepancies that are essential for technology-user satisfaction. Comprehensive training is a generally applicable strategy for mitigating technology-related stress, and early deployment may offset anxiety that exacerbates burnout. Training gaps have been noted in the informatics literature, but have yet to be consistently addressed in practice.^101^^,^^102^

Optimization of HIT, namely EHRs, demonstrated burnout improvement (albeit, some only qualitatively) in all studies identified in this review,^10^^,^^49^^,^^56^^,^^62^^,^^64^^,^^67^ whereas the adoption and/or implementation of EHRs generally had no effect or worsened burnout outcomes.^14^^,^^20^^,^^22^^,^^35^^,^^44^^,^^66^^,^^75^^,^^93^ However, adoption of multiple technologies with interoperability^65^ and the use of EHR-integrated communication^56^ were all effective interventions to increase job satisfaction. Furthermore, based on the evidence obtained from agile methodologies to standardize documentation, there are strong justifications for organizations to tailor EHR usability for precise use cases.^67^ Counterarguments suggest that EHR optimization will not be sufficient as the documentation burden for regulatory purposes (eg, billing and reimbursement) is the primary driver of US dissatisfaction with EHRs.^100^ In consideration with value-based care initiatives, US regulatory changes could potentially lessen the documentation burden by nearly 4-fold.^100^

Until the arrival of policy reform, practical and effective workflow interventions should be leveraged to reduce excessive data entry by the physician. For example, documentation efforts can be shifted from physicians to other members of a care team, which could subsequently improve efficiency. A large body of evidence identified from this review leveraged team-based care, primarily where scribes or medical assistants were added to care teams to document patient encounters in real-time under physician supervision. Expansion of care teams to include scribes has improved efficiency, productivity, quality of patient interactions, and physician satisfaction along with increased revenue and patient satisfaction.^43^^,^^86^ Often coupled with expanded care teams to share documentation tasks, QI initiatives or “lean” methodologies were executed to improve productivity and workflow efficiency related to technologies, improving burnout and/or its proxy measures. The highest-quality study from this category observed a significant reduction in physician burnout following the implementation of QI initiatives that related to routine preventive screening processes and medication reconciliation for chronic disease management.^59^ Notably, burnout was more likely to improve when workflow redesign was part of a QI project where physicians’ concerns were targeted.

An additional burden of the digital environment is the vast amount of information (eg, clerical, medical care, and communication) that physicians are required to handle effectively. Studies that leveraged the expansion of the care team, including scribes, could successfully manage and monitor inbox-related communication including patient portals, and refills and results management. Limited evidence was identified whereby advancements in artificial intelligence (AI), including machine learning, augmented the physician’s management of electronically generated information. Only four studies leveraged clinical decision support and speech recognition algorithms to augment the user and the tool’s performance.^22^^,^^49^^,^^62^^,^^75^ These studies had mixed results on burnout improvement, but improved perceptions of workload^22^ and decreased cognitive workload.^62^ Since information overload is a ubiquitous challenge created by digital tool adoption, this study identifies a clear gap in evaluation of AI solutions—moving beyond accuracy to ascertain what impact, if any, they have on physician workflow, productivity, and burnout.

This study identified a large gap in informatics studies that assess the impact of advanced technologies on physician burnout. Advanced technology applications can support deeper insights, optimized processes, and increased engagement, which can enable greater scale and agility, improving healthcare delivery systems that put the right information, in the right hands, at the right time, with less stress on the care team. With the improved user-centered design by physicians, technology could become an additional member of the care team. Opportunities for automation exist to utilize digital scribing, an automated clinical documentation system that can capture and notate information from the patient–physician clinical encounter;^103^ further integration and implementation of clinical decision support systems to provide information retrieval, evidence-based knowledge, summarizations, and recommendations; the utilization of chatbots, embodied conversational agents, or other virtual assistants to aid task management. In addition to advanced analytics, organizations can further improve interoperability at an operational level with application programming interfaces (APIs) and blockchain, as health data interoperability remains a problem. There remain challenges in patient information exchange between EHRs which further exacerbate physicians’ struggles with data management.

### Strengths and limitations

This systematic review has several strengths. First, this study includes a recently updated systematic review of workplace interventions to tackle burnout with the first focused analysis of burnout specifically related to digital technologies. Secondly, an exhaustive literature search was conducted, including grey literature evaluation, that prioritized sensitivity over specificity. In this update spanning October 2018 to–June 2020, an additional 4173 articles were identified by our search query indicating the increased relevance and prioritization of this topic with 31 newly included studies. Thirdly, the limited evidence from the subset of literature identified gaps in adequately designed and reported studies to examine digital tool usability and its effect on physician burnout. Lastly, the findings from this systematic review support recommendations by the National Academy of Medicine, including the reduction of administration burden and technology optimization, for system-wide actions to reduce clinician burnout.^104^

The results of this systematic review should be interpreted in the context of its limitations. Due to study heterogeneity, it is not possible to provide an assessment of comparative effectiveness across or among interventions (ie, meta-analysis was not possible), and the Oxford Levels of Evidence appraisal tool was used to compare the quality of the evidence provided within a wide variety of study designs. This study only addressed the interventions for burnout on physicians, while digital technologies also contribute to additional work and stress for a wide variety of clinicians, such as nurses and pharmacists. The volume of evidence identified was substantial for physicians alone, and it is likely that mitigation strategies for other healthcare personnel with different work tasks and workflows will vary significantly. This study examined the impact of organization-directed workplace interventions on physician burnout, but it also be must acknowledged that those physicians may be receiving concurrent individualized burnout reduction interventions. Notably, a minority of the studies targeted trainees and most (5 of 7)^17^^,^^41^^,^^53^^,^^56^^,^^57^ interventions were successful;^17^^,^^41^^,^^53^^,^^56^^,^^57^^,^^62^^,^^75^ however, these populations may have different needs to reduce burnout. Most studies were conducted in US primary care settings, which limits generalizability across specialties and globally. Additionally, most studies were of low quality with short duration of follow-up limiting the credibility of the evidence. In this revised protocol to include a subgroup analysis, more details were abstracted and, as a result, some of the 4Ts categorizations changed from the original study such that there were some instances where single categorizations changed to multiple in this update (eg, Contratto, 2016).^29^ Eleven of the studies included in the subgroup analysis were conference abstracts and abstraction details were limited. When assessing the impact of the interventions, only 12 studies with statistical results should be used to compile summation of meaningful positive burnout outcomes—most studies did not conduct statistical analyses or only provided qualitative findings. Many lean methodologies were identified to examine the digital environment efficiency and impact on workflows, but few captured relevant burnout outcomes to meet inclusion criteria.

## CONCLUSION

Interventions designed to optimize technologies, training, and workflows may shift physician burnout to resilience. Factors that contribute significantly to the burden of the digital environment in healthcare include clinical specialty, practice setting, requirements for compliance and reimbursement, and how physicians spend their time. EHR optimization is an effective strategy for mitigating physician burnout, but EHR implementations alone do not improve and may worsen burnout. Comprehensive and appropriately timed training can reduce the stress associated with the introduction of new technologies, including EHRs, but often is not provided. Improvements to address burnout also need to need to consider the larger ecosystem including the organization, the marketplace, and regulatory policies. Workflow redesign and lean methodologies can also be leveraged to reduce the time physicians spend using digital tools and to shift these responsibilities to other care team members. These interventions can improve efficiency and job satisfaction. This study has identified several strategies that can mitigate burnout for physicians; additional research is needed to address the impact of digital tools for other types of clinicians in healthcare settings.

This study is an updated systematic review and subgroup analysis of a previously published systematic review.^10^ From top to bottom description, the primary inclusion criteria required: 1) the study to provide examination of licensed physicians at any career level (eg, trainees and attendings), field (eg, primary, secondary, or mixed) and practice setting (eg, private, academic, government), and 2) study outcomes had to report at least 1 burnout or burnout-proxy measure. Burnout included overall burnout, emotional exhaustion, cynicism, depersonalization, and lack of personal accomplishment. Burnout-proxy measures included satisfaction (eg, physician satisfaction, job satisfaction, joy of practice, and well-being) and stress (eg, general stress, psychological strain, and job distress). Studies had to provide comparisons with no interventions, whereby interventions were limited to organization-directed interventions that related to work, the workplace, or workflow and were categorized into 1 or multiple categories of the 4Ts framework: technology, time, teamwork, and transitions. Technology referred to the implementation or improvement of health information technology, namely EHRs. Time studies involved duty hour restrictions and changes to work schedules or use of time on duty (eg, a program for mindfulness during on-duty time). Teamwork regarded the examination of care team processes and the addition of scribes to the team. Transitions included process improvements and quality initiatives. To identify studies that targeted electronic tool burden and/or its workflow inefficiencies, a subgroup analysis of the identified 4Ts interventions was conducted. The objective of this subgroup analysis was to determine successful interventions that lessened (ie, reduce burnout, improve satisfaction, or decrease stress for physicians) the burden of the digital environment.

Disposition of articles identified from database search queries, the grey literature, and hand searches of included studies, including tracking of articles through the screening phases with reasons provided for full-text exclusions and number of included studies.

The size of each bubble represents the number (n) of studies identified for each 4Ts intervention type—time (light blue), teamwork (medium blue), transitions (dark blue), and technology (navy). Notably, studies were commonly stratified to more than 1 intervention categorization. The x-axis represents the proportion of studies with a positive impact on burnout or 1 of its proxy measures of stress and/or satisfaction. The y-axis represents the proportion of studies designated as high-quality (IA, IIA, or IIB) by the Oxford Levels of Evidence.^13^

## CONSENT FOR PUBLICATION

All authors have approved this manuscript content for submission.

## FUNDING

This research study was supported by IBM Watson Health.

## AUTHOR CONTRIBUTIONS

Conceptualization: KJTC, GPJ, KR. Formal analysis: KJTC, VCW. Methodology: KJTC. Project administration, supervision: KJTC. Validation: KJTC, VCW. Writing original draft: KJTC, VCW, DG, GPJ. Review and editing: all authors.

## ETHICS APPROVAL

No human participants were involved in the study.

## DATA AVAILABILITY

The data underlying this article are available in the article and in its online supplementary material.

## SUPPLEMENTARY MATERIAL

Supplementary material is available at *Journal of the American Medical Informatics Association* online.

## Supplementary Material

ocaa301_Supplementary_Data
