# Supplementary material for: The burden of the digital environment: a systematic review on organization-directed workplace interventions to mitigate physician burnout
Source: J Am Med Inform Assoc. 2021 Jan 19;28(5):985–97. doi: 10.1093/jamia/ocaa301 (PMC8068437; doi:10.1093/jamia/ocaa301)
Supplement: ocaa301_Supplementary_Data [file ocaa301_supplementary_data.docx]

Supplementary Materials

**The burden of digital environment: a systematic review on organization-directed workplace interventions to mitigate physician burnout**

Contents:

- Tables 1-3. Updated Search Strategies across MEDLINE, Cochrane Library, and Embase Databases conducted June 2020
- Tables 4-9. Original Search Strategies across MEDLINE, Cochrane Library, and Embase Databases conducted October 2018
- Table 10. Search Strategy in Association for Computing Machinery (ACM) Digital Library conducted August 2020
- Table 11. Inclusion/Exclusion Criteria
- Table 12. Decision Matrix for Full-Text Screening
- Table 13. Data Extraction Tool
- Table 14. Study Characteristics of All Included Studies (n=81) by Intervention Type
- Table 15. Study Characteristics of Subgroup Analysis (n=38) by Intervention Type

Table 1. UPDATED MEDLINE Search (via PubMed) with “Lean”

| **Search No.** | **Facet** | **Search Terms** | **Search Results**  **(June 1, 2020)** |
| --- | --- | --- | --- |
| 1 | Find studies regarding physicians | physician*[tiab] | 389,692 |
| 2 | Find studies regarding burnout and  stress | burnout, professional[MeSH] | 11,773 |
| 3 |  | ((“burnout” OR "job stress" OR "work engagement" OR "psychosocial factors" OR "health outcomes" OR "work behaviors" OR "job performance" OR "job satisfaction" OR "job-person fit" OR "organizational factors" OR “quadruple aim”)) | 111,338 |
| 4 |  | “stress” | 920,308 |
| 5 |  | ((#2 OR #3 OR #4)) | 1,012,130 |
| 6 | Find studies regarding physicians and burnout or stress | ((#1 AND #5)) | 16,880 |
| 7 | Find studies regarding workflow interventions including “lean” | work flow[MeSH Terms] | 5,029 |
| 8 |  | (("work flow"[tiab] OR workflow[tiab])) | 20,656 |
| 9 |  | ((time and motion studies[MeSH Terms])) | 4,511 |
| 10 |  | Search (“lean”) | 39,264 |
| 11 |  | ((#7 OR #8 OR #9 OR #10)) | 66,355 |
| 12 | Find studies regarding physicians, burnout, and workflow interventions | (#6 AND #11) | 43,842 |
| 13 | Studies published after October 2018 to update search | #12 AND (2018/10:2020/06 [edat]) | 3,795 |
| 14 | Find and exclude case reports | case reports[pt] | 2,099,057 |
| 15 |  | #13 NOT #14 | 3,778 |
| 16 | Find and exclude narrative reviews | Review[pt] NOT (Cochrane OR systematic or meta-analy*) | 2,356,693 |
| 17 |  | #15 NOT #16 | 3,587 |
| 18 | Find and exclude non-human studies | (#17 AND Humans[MeSH:noexp]) OR (#17 NOT animals[MeSH:noexp]) | 3,371 |
| 19 | Limit to studies with abstracts | #18 Filters: Abstract | 3,309 |
| 20 | Limit to studies published in English | #18 Filters: Abstract; English | 3,279 |

Table 2. UPDATED Cochrane Library Search with “Lean”

| **Search No.** | **Facet** | **Search Terms** | **Search Results**  **(June 3, 2020)** |
| --- | --- | --- | --- |
| 1 | Find studies regarding physicians | “physician*” | 42,135 |
| 2 | Find studies regarding burnout and  stress | burnout, professional[MeSH] | 252 |
| 3 |  | ((“burnout” OR “job stress” OR “work engagement” OR “psychosocial factors” OR “health outcomes” OR “work behaviors” OR “job performance” OR “job satisfaction” OR “job-person fit" OR "organizational factors" OR “quadruple aim”)) | 85,546 |
| 4 |  | “stress” | 575,574 |
| 5 |  | ((#2 OR #3 OR #4)) | 134,264 |
| 6 | Find studies regarding physicians and burnout or stress | ((#1 AND #5)) | 7,101 |
| 7 | Find studies regarding workflow interventions including “lean” | work flow[MeSH Terms] | 66 |
| 8 |  | (("work flow"[tiab] OR workflow[tiab])) | 868 |
| 9 |  | ((time and motion studies[MeSH Terms])) | 156 |
| 10 |  | Lean[MeSH] | 138 |
| 11 |  | ((#7 OR #8 OR #9 OR #10)) | 1,149 |
| 12 | Find studies regarding physicians, burnout, and workflow interventions | (#6 AND #11) | 97 |
| 13 | Studies published in “Trials” after October 2018 to update search | #12 AND (2018/11:2020/06)/limit | 47 |

Table 3. UPDATED Embase Search with “Lean”

| **Search no.** | **Facet** | **Search Terms** | **Search Results**  **(June 3, 2020)** |
| --- | --- | --- | --- |
| 1 | Find articles on physicians | ‘physician’/exp OR ‘physician*’:ab,ti | 1,148,919 |
| 2 | Find articles on job burnout | ‘burnout’ OR ‘job stress’ OR ‘work engagement’ OR ‘psychosocial factors’ OR ‘health outcomes’ OR ‘job performance’ OR ‘job satisfaction’ OR ‘work behavior*’ OR ‘job-person fit’ OR ‘organizational factors’ OR ‘quadruple aim’ | 162,049 |
| 3 |  | ‘stress’ | 1,282,014 |
| 4 |  | ‘professional burnout’ OR ‘burnout’/exp | 18,328 |
| 5 |  | #2 OR #3 OR #4 | 1,411,609 |
| 6 | Find articles on physicians and burnout | #1 AND #5 | 50,254 |
| 7 | Find articles on workflow | ‘workflow’/exp | 22,042 |
| 8 |  | ‘workflow’:ti,ab OR ‘work flow’:ti,ab | 31,324 |
| 9 |  | ‘time and motion studies’:ti,ab OR ‘task performance’/exp | 143,402 |
| 10 |  | ‘lean’ | 60,125 |
| 11 |  | #7 OR #8 OR #9 or #10 | 238,655 |
| 12 | Combine article on physicians and burnout with ‘Lean’ not in other articles about ‘workflow’ | #6 AND #11 | 648 |
| 13 | Studies published after October 2018 to update search | #12 AND [10-3-2018]/sd NOT [6-03-2020]/sd | 170 |
| 14 | Find and exclude articles in PubMed | #13 AND [embase]/lim | 150 |
| 15 | Find and exclude case reports | #14 NOT ‘case report’/de | 145 |
| 16 | Exclude non-primary studies (except systematic reviews) | #15 NOT (‘editorial’/it OR ‘letter”/it OR ‘note’/it or ‘secondary analysis’/de) | 136 |
| 17 | Find and exclude non-human studies | #16 NOT (‘animal experiment’/de OR ‘animal model’/de OR ‘nonhuman’/de) | 134 |
| 18 | Limit to studies with abstracts | #17 AND [abstracts]/lim | 128 |
| 19 | Limit to studies published in English | #18 AND [English]/lim | 128 |

NOTE: In Tables 1-3, the search query used the exact same terms (both MeSH and keywords) as the original searches, but applied a publication date filter to capture the studies published between October 2018 and June 2020. One difference is that the original study had a secondary search query (as recommended through the peer-review process) to explicitly search for “lean” methodologies. Rather than performing multiple update search queries, lean” was added to the workflow interventions search string facet. As such, one additional line of Boolean logic was removed (i.e., find lean interventions, but exclude other workflow interventions) compared to the original search and in doing so the scope was increased by the removal of this logic making the search more specific, but less sensitive.

Table 4. MEDLINE Search (via PubMed)

| **Search No.** | **Facet** | **Search Terms** | **Search Results**  **(October 3, 2018)** |
| --- | --- | --- | --- |
| 1 | Find studies regarding physicians | physician*[tiab] | 357,549 |
| 2 | Find studies regarding burnout and  stress | burnout, professional[MeSH] | 9,912 |
| 3 |  | ((“burnout” OR "job stress" OR "work engagement" OR "psychosocial factors" OR "health outcomes" OR "work behaviors" OR "job performance" OR "job satisfaction" OR "job-person fit" OR "organizational factors" OR “quadruple aim”)) | 93,009 |
| 4 |  | “stress” | 814,346 |
| 5 |  | ((#2 OR #3 OR #4)) | 890,903 |
| 6 | Find studies regarding physicians and burnout or stress | ((#1 AND #5)) | 14,608 |
| 7 | Find studies regarding workflow interventions | work flow[MeSH Terms] | 3,311 |
| 8 |  | (("work flow"[tiab] OR workflow[tiab])) | 15,246 |
| 9 |  | ((time and motion studies[MeSH Terms])) | 4,198 |
| 10 |  | ((#7 OR #8 OR #9)) | 20,809 |
| 11 | Find studies regarding physicians and burnout and workflow interventions | ((#6 AND #10)) | 74 |

**Table 5. Cochrane Library Search**

| **Search No.** | **Facet** | **Search Terms** | **Search Results**  **(October 3, 2018)** |
| --- | --- | --- | --- |
| 1 | Find articles on physicians | Physician* | 37,421 |
| 2 | Find articles on burnout or job performance | Burnout, Professional [MeSH] | 186 |
| 3 |  | “burnout” OR “job stress” OR “work engagement” OR “psychosocial factors” OR “health outcomes” OR “work behaviors” OR “job performance” OR “job satisfaction” OR “job-person fit” OR “organizational factors” OR “stress” | 48,759 |
| 4 |  | #2 OR #3 | 48,759 |
| 5 | Combine articles on physicians and burnout | #1 AND #4 | 2,333 |
| 6 | Find articles on workflow | Workflow [MeSH] | 46 |
| 7 |  | “Work flow” OR “workflow” OR “time and motion studies” | 744 |
| 8 |  | Time and Motion Studies [MeSH] | 123 |
| 9 |  | #6 OR #7 OR #8 | 744 |
| 10 | Combine articles on physician burnout and workflow | #5 AND #9 | 17 |

**Table 6. EMBASE Search**

| **Search No.** | **Facet** | **Search Terms** | **Search Results**  **(October 3, 2018)** |
| --- | --- | --- | --- |
| 1 | Find articles on physicians | ‘physician’/exp OR ‘physicians’:ab,ti | 862,826 |
| 2 | Find articles on job burnout | ‘burnout’/exp OR ‘burnout’:ab,ti OR ‘job stress’/exp OR ‘job stress’:ab,ti OR ‘work engagement’/exp OR ‘work engagement’:ab,ti OR ‘psychosocial factors’/exp OR ‘psychosocial factors’:ab,ti OR ‘health outcomes’/exp OR ‘health outcomes’:ab,ti OR ‘job performance’/exp OR ‘job performance’:ab,ti OR ‘job satisfaction’/exp OR ‘job satisfaction’:ab,ti OR ‘stress’/exp OR ‘stress’:ab,ti OR ‘work behavior’:ab,ti OR ‘job-person fit’:ab,ti OR ‘organizational factors’:ab,ti | 1,015,043 |
| 3 | Find articles on physicians and burnout | #1 AND #2 | 33,047 |
| 4 | Find articles on workflow | ‘workflow’/exp OR ‘workflow’:ab,ti OR ‘work flow’:ab,ti OR ‘task performance’/exp OR ‘time and motion studies’:ab,ti | 161,572 |
| 5 | Combine articles on physicians’ burnout and workflow | #3 AND #4 | 389 |
| 6 | Find and limit conference abstracts to last three years | [conference abstract]/lim NOT [2017-2018]/py | 2,597,920 |
| 7 |  | #5 NOT #6 | 326 |

**Table 7.** **Additional MEDLINE Search (via PubMed) with “Lean”**

| **Search No.** | **Facet** | **Search Terms** | **Search Results**  **(October 3, 2018)** |
| --- | --- | --- | --- |
| 1 | Find studies regarding physicians | Search physician*[tiab] | 357,549 |
| 2 | Find studies regarding burnout and  stress | Search burnout, professional[MeSH] | 9,912 |
| 3 |  | Search ((“burnout” OR "job stress" OR "work engagement" OR "psychosocial factors" OR "health outcomes" OR "work behaviors" OR "job performance" OR "job satisfaction" OR "job-person fit" OR "organizational factors" OR “quadruple aim”)) | 93,009 |
| 4 |  | Search stress | 814,346 |
| 5 |  | Search ((#2 OR #3 OR #4)) | 890,903 |
| 6 | Find studies regarding physicians and burnout or stress | Search ((#1 AND #5)) | 14,608 |
| 7 | Find studies regarding workflow interventions | Search work flow[MeSH Terms] | 3,311 |
| 8 |  | Search (("work flow"[tiab] OR workflow[tiab])) | 15,246 |
| 9 |  | Search ((time and motion studies[MeSH Terms])) | 4,198 |
| 10 |  | Search ((#7 OR #8 OR #9)) | 20,809 |
| 11 | Find studies regarding Lean interventions, excluding other workflow interventions | Search (lean NOT #10) | 35,392 |
| 12 | Find studies regarding physicians, burnout, and Lean interventions | Search (#6 AND #11) | 13 |

Table 8. Additional Cochrane Library Search with “Lean”

| **Search no.** | **Facet** | **Search Terms** | **Search Results**  **(October 3, 2018)** |
| --- | --- | --- | --- |
| 1 | Find articles on physicians | “Physician*” | 37,421 |
| 2 | Find articles on job burnout | “Burnout, professional” [MeSH] | 186 |
| 3 |  | “burnout” OR “job stress” OR “work engagement” OR “psychosocial factors” OR “health outcomes” OR “work behaviors” OR “job performance” OR “job satisfaction” OR “job-person fit” OR “organizational factors” OR “quadruple aim” | 7,478 |
| 4 |  | “Stress” | 42,766 |
| 5 |  | #2 OR #3 OR #4 | 48,759 |
| 6 | Find articles on physicians and job burnout | #1 AND #5 | 2,333 |
| 7 | Find articles on workflow | “workflow”[MeSH] | 46 |
| 8 |  | “work flow” OR “workflow” | 628 |
| 9 |  | “Time and Motion Studies” [MeSH] | 123 |
| 10 |  | #7 OR #8 OR #9 | 742 |
| 11 | Find articles on “lean” | “lean” | 5,774 |
| 12 | Find articles on ‘Lean’ not in other articles about ‘workflow’ | #11 NOT #10 | 5,768 |
| 13 | Combine article on physicians and burnout with ‘Lean’ not in other articles about ‘workflow’ | #6 AND #12 | 41 |

**Table 9. Additional Embase Search with “Lean”**

| **Search no.** | **Facet** | **Search Terms** | **Search Results**  **(October 3, 2018)** |
| --- | --- | --- | --- |
| 1 | Find articles on physicians | ‘physician’/exp OR ‘physician*’:ab,ti | 879,438 |
| 2 | Find articles on job burnout | ‘burnout’ OR ‘job stress’ OR ‘work engagement’ OR ‘psychosocial factors’ OR ‘health outcomes’ OR ‘job performance’ OR ‘job satisfaction’ OR ‘work behavior*’ OR ‘job-person fit’ OR ‘organizational factors’ OR ‘quadruple aim’ | 124,809 |
| 3 |  | Stress | 1,043,526 |
| 4 |  | ‘professional burnout’ OR ‘burnout’/exp | 12,688 |
| 5 |  | #2 OR #3 OR #4 | 1,143,835 |
| 6 | Find articles on physicians and burnout | #1 AND #5 | 37,053 |
| 7 | Find articles on workflow | ‘workflow’/exp | 12,182 |
| 8 |  | ‘workflow’:ti,ab OR ‘work flow’:ti,ab | 19,356 |
| 9 |  | ‘time and motion studies’:ti,ab OR ‘task performance’/exp | 130,822 |
| 10 |  | #7 OR #8 OR #9 | 152,627 |
| 11 | Find articles on ‘Lean’ not in other articles about ‘workflow’ | ‘lean’ NOT #10 | 49,633 |
| 12 | Combine article on physicians and burnout with ‘Lean’ not in other articles about ‘workflow’ | #6 AND #11 | 47 |

Table 10. The ACM Guide to Computing Literature Search

| **Search no.** | **Facet** | **Search Terms** | **Search Results**  **(August 17, 2020)** |
| --- | --- | --- | --- |
| 1 | Find articles on physicians | [abstract: physician*] | 4,470 |
| 2 | Find articles on job burnout | all: [“job stress” OR “work engagement” OR “psychosocial factors” OR “health outcomes” OR “work behavior” or “job performance” OR “job satisfaction” OR “job-person fit” OR “organizational factors” OR “quadruple aim”] | 10,430 |
| 3 | Find articles on physician burnout | #1 AND #2 | 132 |
| 4 | Find articles on workflow including lean | all: [“time and motion” OR “lean” OR “lean method” OR “workflow” or “work flow”] | 9,625 |
| 5 | Combine articles on physician burnout and workflow including lean | #3 AND #4 | 49 |
| 6 | Limit to publication timeframe January 2007 – June 2020 | [publication date: (01/01/2007 TO 6/30/20200] | 46 |

**Table 11. Inclusion/Exclusion Criteria**

| **PICOST** | **Inclusion** | **Exclusion** |
| --- | --- | --- |
| Population | The publication pertains to **physicians** (including medical specialties classified according to surgical or internal medicine, age range of patients, diagnostic or therapeutic, and organ-based or technique-based specialists). | The publication does not pertain to physicians or pertains to solely non-physician healthcare professionals (e.g. nurses, assistants, trainees, caregivers at nursing homes, etc.).  Aggregated data will be excluded, subgroup analysis will be required for physicians. |
| Intervention: Part I | The publication reports on **organization-directed interventions** that are related to work, the workplace, or workflow. | The publication does not report on organization-directed interventions that are related to work, the workplace, or workflow.  Physician-directed interventions for self-care (building individuals’ resilience outside of work) will be excluded. However, if time and/or training to improve self-care is provided to physicians by the organization the study will be included. |
| Intervention:  Part II  (subgroup analysis) | The publication reports on interventions that address **electronic tool burden and/or its workflow inefficiencies**. | The publication reports on other interventions that are not related to the use of technology or improving workflows. |
| Comparison | The publication compares an intervention versus no intervention. | NA |
| Outcomes | The publication reports on at least one of these measures -   1. **Burnout** including overall burnout, emotional exhaustion, cynicism, depersonalization, and lack of personal accomplishment. 2. **Satisfaction** including physician satisfaction, job satisfaction, joy of practice, and well-being. 3. **Stress** including (general) stress, psychological strain, and job distress. | The publication does not report on burnout, satisfaction, or stress.  Mental health outcomes including anxiety and depression will be excluded. |
| Setting | Any practice setting will be included (primary, secondary, mixed, or unspecified). | None. |
| Time | The study is published within the last 13 years (2007 – 2020). | The study is before 2007. |
| Limits | The publication is in English. | The publication is not in English. |
|  | The publication reports a primary study or systematic review with or without a meta-analysis using similar inclusion criteria. | The publication reports a narrative (non-systematic) review, case report, or case series. Secondary studies will be excluded. Systematic reviews with other inclusion criteria. |
|  | The publication contains an abstract. | The publication does not contain an abstract. |

**Table 12. Decision Matrix for Full-Text Screening**

| **DistillerSR Screening Form** |
| --- |

**
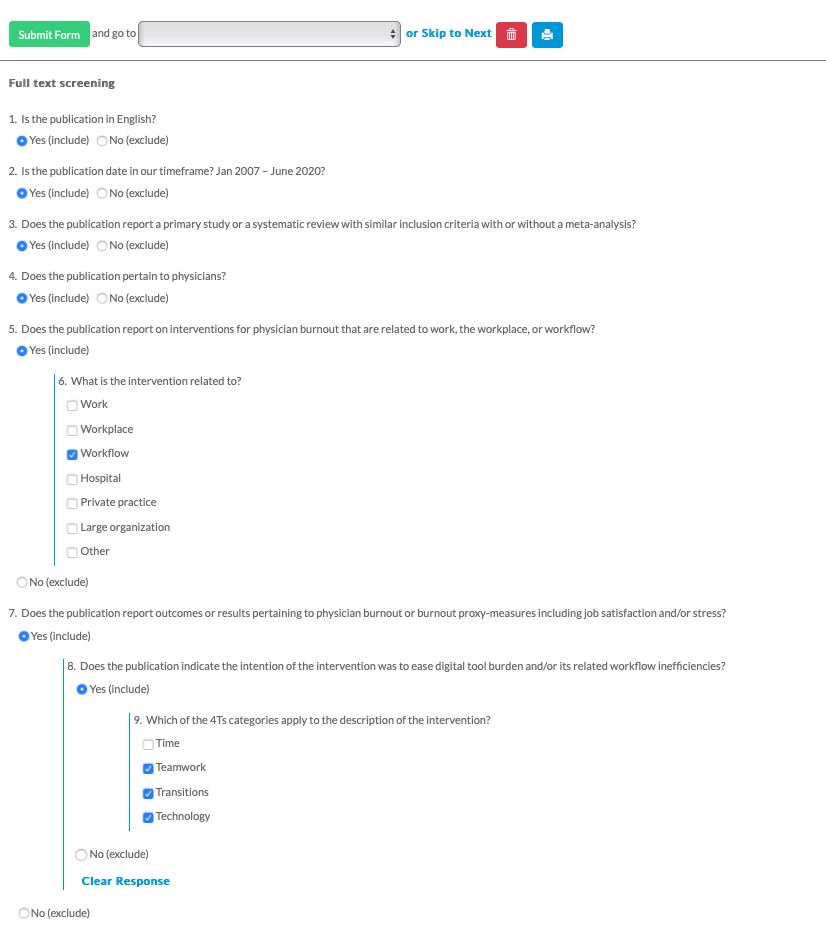
**

**Table 13. Data Extraction Tool**

| **DistillerSR Data Extraction Form** |
| --- |


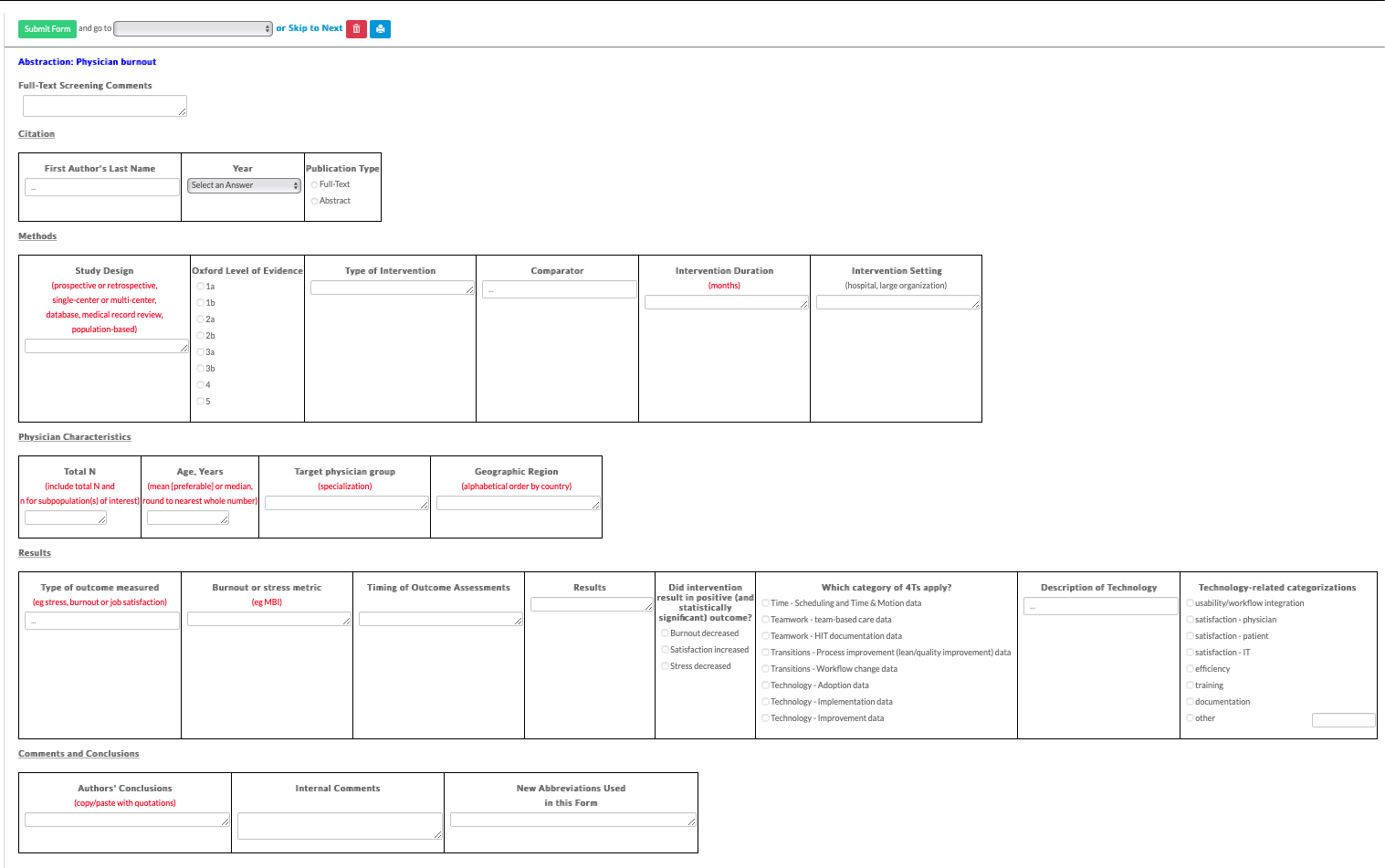


**Supplementary Table 14. Study characteristics of all included (n=81) studies by 4Ts (time, teamwork, transitions, technology) intervention type**

| **Reference** | **Country** | **Study design** | **N** | | **Population and setting** | | **Type of intervention** | **Outcome** | | **Follow-up** | | **Level of evidence^a^** |
| --- | --- | --- | --- | --- | --- | --- | --- | --- | --- | --- | --- | --- |
| ***Time* (N=56)** | | | | | | | | | | | | |
| Ali, 2011[1] | US | Cluster RCT | 45 | | Physicians with various specialties   ICU | | **Time:** Two intensivist staffing schedules were compared: continuous and interrupted (rotations every 2 wk) for 14 mo | Scales derived from the National Study of the Changing Workforce | | 9 mo | | IB |
| Amis, 2018[2]^d^ | UK | Pre-post intervention survey | 13 | | First year residents | | **Time:** A checklist aimed to reduce the number of inappropriate prescribing tasks | Job satisfaction | | 3 weekends | | IV |
| Amutio, 2015[3] | Spain | Pre-post intervention survey | 42 | | Physicians  NR | | **Time:** Provided work time for 8 weekly mindfulness-based stress reduction (MBSR) sessions of 2.5 h each plus one 8 h retreat. | MBI | | 10 mo | | IB |
| Ares, 2019[4]^c^ | US | Pre-post intervention survey | 28 | | Neurosurgery residents  NR | | **Time:** Resident wellness program to focus on physical and mental health that included monthly wellness lecture series, free access to hospital gym, group gym visits and mentorship | MBI | | 1 yr | | IV |
| Baccei, 2020[5]^c,d^ | US | Pre-post intervention survey | 6 | | Musculoskeletal radiologists  Academic medical center hospital and outpatient location | | **Time:** Lean methodology as part of quality improvement initiative in radiology department that included work schedule modifications and processes to decrease turnaround time of results | Custom satisfaction survey | | 6 mo | | IV |
| Bragard, 2010[6] | Belgium | RCT | 96 | | Oncology residents  NR | | **Time:** Resident program to improve stress management. Provided 30 h communication skills training and 10 h stress management skills training in small groups | MBI; Stress visual analog scales | | 8 mo | | IB |
| Butow, 2008[7] | Australia | RCT | 35 | | Oncologists  Six teaching hospitals in different cities | | **Time:** Provided work time for 1.5 d workshop on communication skills training with 3-6 participants. Concentrated on collaborative framework, blocking, and responding to emotions like anxiety, depression, and anger | MBI | | 12 mo | | IB |
| Butow, 2015[8] | Multinational | RCT | 62 | | Oncologists  Large oncology centers | | **Time:** Provided 7 h workshop on shared decision-making framework, structuring information for patients, ensuring key information is communicated clearly, and avoiding coercive communication were all taught and practiced in the workshop | MBI | | 1 mo | | IB |
| Contratto, 2016[9]^c,d^ | US | Pre-post intervention survey | 9 | | Primary care physicians  Urban academic general internal medicine primary care practice | | **Time:** Evaluated documentation effort following a workflow change including full-time clerical support to enter tests ordered by physicians, identify incomplete health maintenance measures, and preload new patient information | 14-item validated survey including burnout and subscale of depersonalization | | 4 mo | | IV |
| Contratto, 2017[10]^c,d^ | US | Quasi-experimental (single group pre-post intervention) mixed-methods | 7 | | Internal medicine physicians  Academic general internal medicine practice | | **Time:** Modified workflow to include clerical support personnel for physician order entry and examined productivity | Burnout: MBI; Satisfaction | | 4 mo | | IV |
| DeChant, 2019[11]^c,d,e^ | Multinational | Systematic review | NA | | NA | | **Time:** Varied | Varied | | Varied | | IV |
| Desai, 2018[12] | US | Cluster-randomized trial | 80 | | First-year residents  63 internal medicine residency practices | | **Time:** Duty-hour policies of the 2011 Accreditation Council for  Graduate Medical Education (ACGME) | Overall well-being; MBI | | 7 mo | | IB |
| Dyrbye, 2016[13]^c^ | US | RCT | 290 | | Internal medicine and surgery specialists  Departments in two states | | **Time:** Organization designated tasks and provided business time to cultivate well-being in job satisfaction, teamwork, relationships, personal strengths, problem solving, and positive emotions | MBI; Work Engagement Scale; Empowerment at Work Scale; Physician JSS | | 3 mo | | IB |
| Dyrbye, 2019[14] | US | RCT | 88 | | General internal medicine, family medicine, and general pediatrics physicians  Multi-site urban health system | | **Time:** Provided professional coaching sessions (3.5 h) by telephone | MBI; 12-item Global Job Satisfaction subscale of the Physician JSS | | 17 mo | | IB |
| Dunn, 2007[15]^c,d^ | US | Noncontrolled prospective intervention study | 22-32 | | Physicians  Primary care group | | **Time:** Data guided interventions and systematic improvement processes that included providing schedule flexibility | ACP/ASIM survey on physician satisfaction; MBI; QWC | | 6 yr | | IV |
| Ehrlich, 2016[16]^e^ | US | Pre-post intervention survey | 25 | | Ophthalmologists  Large academic ophthalmology department | | **Time:** Examined documentation time, efficiency, and workflow after EHR system implementation | 30 question survey using Likert scale rating; Job satisfaction | | 24 mo | | IV |
| Fassiotto, 2016[17] | US | Pre-post intervention survey | 60 | | Medical school faculty  NR | | **Time:** Time-banking intervention measures unacknowledged teaching, service and clinical activities, and acknowledges them with practical rewards | NR | | Post intervention | | IV |
| Garland, 2012[18] | Canada | Crossover RCT | 34 | | Physicians  ICU | | **Time:** Shift work staffing in which there was 24/7 intensivist presence. The same pool of intensivists supplied day shift and night shit coverage. In any given week, a single intensivist was responsible for all 7-day shifts, whereas 2 different intensivists alternated the 7 nightshifts | MBI (emotional exhaustion subscale) | | Post intervention | | IB |
| Gidwani, 2017[19]^b,c^ | US | RCT | 4 | | Physicians  Academic family medicine clinic | | **Time:** Scribes used to draft all relevant documentation and examined charting time and efficiency | Physician satisfaction, measured by a 5-item instrument that included physicians’ perceptions of chart quality and chart accuracy | | 1 yr | | IB |
| Goodman, 2012[20] | US | Pre-post intervention survey | 51 | | Physicians  NR | | **Time:** Organization sponsored mindfulness-based stress reduction courses including 7 h silent retreat | MBI; SF-12 Health Survey version 2 | | 8 w | | IV |
| Goyal, 2018[21]^d,e^ | US | Prospective cohort | 7 | | General internal medicine physicians (attendings, supervising residents, first-year residents)  Major urban academic health center | | **Time:** Provided training and implementation of nurse-physician alphanumeric paging system to enhance communication and decrease frequency of disruptions | NR | | 2 w | | IIB |
| Hart, 2019[22] | US | Pre-post intervention survey | 46 | | Emergency medicine residents  Urban hospital | | **Time:** Corporate wellness program (“The Happiness Practice”), completed monthly 1 h didactic sessions during work hours | MBI | | 6 mo | | IV |
| Heaton, 2016[23]^c^ | Multinational | Systematic review | NA | | NA | | **Time:** Scribes used for documentation and examined throughput in workflow | Provider satisfaction | | NA | | IIa |
| Imdieke, 2017[24]^c,d^ | US | Quasi-experimental, nonrandomized  pre- and post-intervention study | 2 | | Internal medicine physicians  Hospital-based, outpatient primary care clinic | | **Time:** Medical scribes used in an ambulatory clinic to support provider documentation in the EHR and examined documentation time | Provider satisfaction | | 4-6 wk | | IV |
| Ireland, 2017[25] | Australia | RCT | 44 | | Emergency department interns  Major urban hospital | | **Time:** Training program provided 1 h mindfulness-based stress reduction training workshop for 10 w | Copenhagen Burnout Inventory; Perceived Stress Scale | | Post-intervention | | IB |
| Isaksson, 2010[26] | Norway | Pre-post intervention survey | 227 | | Physicians  NR | | **Time:** Organization sponsored either a 6-7 h non-treatment counselling session with a psychiatrist or specialist in occupational medicine to identify work and private factors contributing to stress and recommendation to actively deal with those needs or a 5 d group course for up to 8 participants led by a counsellor and occupational therapist to be completed during work hours | MBI; Cooper Job Stress Questionnaire; Ways of Coping Checklist | | 3 yr | | IV |
| Joseph, 2017[27]^c,e^ | US | Pre-post intervention survey | NR | | Providers  NR | | **Time:** The impact of a brief, intensive technology adoption and training intervention aimed at improving individual clinician’s efficiency in using EHR by examining documentation time | NR | | NR | | IV |
| Kakarala, 2018[28] | US | Pre-post intervention survey | 21 | | Internal medicine program (interns and faculty coaches)  Community-based hospital | | **Time: Resident program i**mplemented coaching by faculty that were trained and certified to be Health and Wellness coaches | MBI | | 1 yr | | IV |
| Keefer, 2018[29]^c,d^ | US | Pre-post intervention survey | NP | | Physicians  Pediatric hospital | | **Time:** Lean methodology to reduce overwork and burden for residents. Additional full-time physicians were hired and schedule modifications were provided to reduce patient-to-attending ratio and added in-house evening and overnight resident supervision | MindTools | | 3 yr | | IV |
| Kim, 2011[30] | US | Prospective cohort | 56 | | Residents  Large pediatric training program | | **Time:** 2003 ACGME work hour limits | NR | | 7 yr | | IV |
| Krasner, 2009[31] | US | Pre-post intervention survey | 70 | | Primary care physicians  Urban settings | | **Time:** Organization provided eight 2.5 h mindfulness training sessions plus one 7 h silent retreat followed by ten 2.5 h monthly sessions | MBI; Jefferson Scale of Physician Empathy; Profile of Mood States | | 15 mo | | IV |
| Landrigan, 2008[32] | US | Prospective cohort | 220 | | Residents  Pediatric residency programs at hospitals | | **Time:** 2003 ACGME work hour limits for resident physicians in the US. Residents can work no more than 30 consecutive hrs and no more than 80 to 88 h/wk, averaged over 4 wks | MBI | | 1 yr | | IV |
| Lapointe, 2018[33]^e^ | US | Pre-post intervention survey | 25 | | Internal medicine residents  591-bed urban hospital | | **Time:** Improved communication using EHR-based text paging system and measured disruptions in time | Stress | | 6 mo | | IV |
| Lucas, 2012[34] | US | Cluster randomized crossover noninferiority trial | 62 | | Physicians  University affiliated teaching hospital | | **Time:** Assignment to random sequences of 2- and 4-week rotations | Questionnaire includes questions from MEMO; perceived stress scale; MBI; national job burnout survey | | 2/4 wks | | IB |
| Martins, 2011[35] | Argentina | RCT | 74 | | Pediatric residents  Tertiary care pediatric hospital | | **Time:** Provided two 2.5 h workshops covering risk factors and tools to cope with burnout | MBI | | Post-intervention | | IB |
| Mazur, 2019[36]^d,e^ | US | Prospective cohort | 38 | | Internal medicine, family medicine, pediatrics, surgery, and other specialty residents and fellows  Large academic institution | | **Time:** Quality improvement process to improve EHR with enhanced interface usability that automatically sorted all previously identified critical test results for patients. Also displayed info regarding patient status and decision support. Determined impact on time for task completion and examined workflow change using decision support | NASA-Task Load Index (Perceived workload and satisfaction) | | Post-intervention | | IIB |
| McCormick, 2018[37]^c,d^ | US | Pre-post intervention survey | 6 | | Urologists  Academic urology clinic | | **Time:** Scribes used for documentation and productivity | Work satisfaction | | 3 mo | | IV |
| Mehta, 2018[38]^c,d,e^ | US | Pre-post intervention survey | NP | | Hospitalists  3 academic medicine sites | | **Time:** Use of scribes, modified EHR-related workflows, and customized EHR templates to reduce documentation time, increase time spent for patient encounters, and improve work-life balance | Custom satisfaction survey | | 6 mo | | IV |
| Milenkiewicz, 2017[39]^e^ | US | Pre-post intervention survey | NR | | Physicians  Department of Addiction Medicine at Kaiser Permanente | | **Time:** Examined usability of an EHR tool to improve and standardize the documentation process including documentation time | NR | | Post- intervention | | IV |
| Milstein, 2009[40] | US | Pre-post intervention survey | 33 | | Pediatric residents  Large urban academic health system | | **Time:** Provided training to use self-administered psychotherapeutic tool "BATHE" adapted from "The Fifteen Minute Hour" | MBI | | 3 mo | | IV |
| Minichiello, 2020[41] | US | Pre-post intervention survey | 17 | | Family medicine residents  Large academic institution | | **Time:** Provided optional 5-session, 10 h mindfulness training program | MBI; Perceived Stress Scale-10; Brief Resilience Scale | | 5 mo | | IV |
| Mishra, 2018[42]^c,d^ | US | Dual-balanced crossover prospective cohort study | 18 | | Primary care providers without experience with scribes  Two medical centers in an integrated network | | **Time:** Assignment of medical scribes for electronic documentation (alternating 3 mo intervals with and without scribe support; randomized to start with or without support) and determine if documentation time is reduced for physicians | 5-point Likert Scale | | 12 mo | | IIB |
| Moeller, 2017[43] | US | Pre-post intervention survey | NR | | Physicians  NR | | **Time:** *Pilot Practice Refresh* program that initially reduces and then gradually increases the time physicians spend with patients so that physicians can learn and practice skills in efficiency, teamwork, and self-care | NR | | NR | | IV |
| Moffat-Bruce, 2019[44] | US | Pre-post intervention survey | 50 | | Residents and faculty  Academic medical center | | **Time:** Organization provided three interventions: 1) Mindfulness training for clinical teams; 8-w program with 1 h/w (8 h), then 1-h monthly booster for 6 mo (6 h); 2) Flipped classroom mindfulness/resilience training consisting of 2 components: 4 online modules and 3 interactive sessions (7 h); 3) Health and Wellness program to support 1 and 2 | MBI; Physician Well-Being Index | | 4 mo | | IV |
| Morrow, 2014[45] | UK | Cross-sectional | 82 | | Junior doctors  Deanery | | **Time:** UK WTR applied fully to junior doctors since 2009, with a limit of 48 h/wk, averaged across a reference period of 26 wks, alongside specified minimum rest periods | NA | | NA | | IV |
| Parshuram, 2015[46] | Canada | RCT | 47 | | Residents  University affiliated ICUs | | **Time:** In house overnight schedules of 24, 16 or 12 h | MBI | | 2 mo | | IIB |
| Ripp, 2015[47] | US | Pre-post intervention survey | 128 (2011-2012 cohort); 111 (2008-2009 cohort) | | Internal medicine residents  Academic medical centers | | **Time:** 2011 ACGME modified duty hours standards to limit continuous duty of first-year residents to 16 h | MBI; ESS | | 1 yr | | IV |
| Runyan, 2016 [48] | US | Pre-post intervention survey | 12 | | Family medicine residents  Three health centers (urban, rural, and federally qualified health center) | | **Time:** Resident program provided wellness curriculum | MBI; Self Compassion Scale; Jefferson Empathy Scale; Perceived Stress Scale | | 3 mo | | IV |
| Schuh, 2011[49] | US | Prospective, unblinded cohort study | 34 | | Neurology residents  Neurology residency program | | **Time:** 2008 Institute of Medicine work duty hour recommendations that limit shifts to 16 or 24 h with a 5 h nap, eliminate averaging of any on-call shifts, increase time off between shifts for night float and overnight call, limit consecutive night float shifts to 4, and provide 1 day off /wk/5 per mo w/out averaging | MBI | | 1 mo | | IV |
| Shea, 2014[50] | US | RCT | 106 | | Graduate internal medicine interns  Internal medicine service hospital | | **Time:** Provided a2-h period of protected time in which interns were expected to sleep (12:30 am to 5:30 am) for 4 wk | MBI | | Post-intervention | | IB |
| Sherlock, 2016[51] | UK | Pre-post intervention survey | 15 | | General practitioners  One urban and semi-rural health authority area | | **Time:** Organization provided 12 fortnightly seminars giving instruction in adaptation practice for coping with stress, anxiety, and depression | Simple Stress Scale | | 6 mo | | IV |
| Sood, 2011[52] | US | RCT | 40 | | Department of medicine physicians  Tertiary care medical center | | **Time:** Organization provided a single 90 min training in Stress Management and Resiliency adapted from Attention and Interpretation Therapy, including paced breathing meditation. Optional 30-60 min follow-up sessions offered after session completion | Connor-Davidson Resilience Scale; Linear Analog Self- Assessment Scale; Visual Analog Scale-Fatigue ;  Perceived Stress Scale (PSS | | 8 w | | IB |
| Tucker, 2010[53] | Wales | Cross-sectional | 336 | | Residents and interns   NR | | **Time:** Schedule design | Questionnaire developed for study | | NA | | IV |
| Verweij, 2016[54] | Netherlands | Pre-post intervention survey | 50 | | General practice trainees  Training hospital | | **Time:** Provided work time for 50% of continuing education practices for mindfulness-based stress reduction | Urtrecht burnout scale for contractual occupations (validated Dutch MBI equivalent ) | | NR | | IV |
| Willard-Grace, 2017[55]^c,d^ | US | Cross-sectional | 236 | | Clinicians  County-run primary care clinics | | **Time:** A defined model of team-based care (enhanced roles for medical assistants, registered nurses, and behavioral health providers) is defined, workflow changed, and schedules redesigned | MBI | | NR | | IV |
| ***Teamwork* (N=30)** | | | | | | | | | | | | |
| Ares, 2019[4]^b^ | US | Pre-post intervention survey | 28 | | Neurosurgery residents  NR | | **Teamwork:** Resident wellness program to focus on physical and mental health that supported mentorship | MBI | | 1 yr | | IV |
| Baccei, 2020[5]^b,d^ | US | Pre-post intervention survey | 6 | | Musculoskeletal radiologists  Academic medical center hospital and outpatient location | | **Teamwork/Transitions:** Lean methodology as part of quality improvement initiative in radiology department that included work schedule modifications for team-based care and modified workflow processes to decrease turnaround time of results | Custom satisfaction survey | | 6 mo | | IV |
| Chapman, 2017[56]^d^ | US | Cross-sectional | 886 | | Primary care practices | | **Teamwork/Transitions:** Employing medical assistants (MA) in an innovative model of care with workflow modifications for documentation | Satisfaction interview | | 4 yr | | IV |
| Contratto, 2016[9]^b,d^ | US | Pre-post intervention survey | 9 | | Physicians  Urban academic general internal medicine primary care practice | | **Teamwork/Transitions:** To evaluate the impact of modifying workflow to include full-time clerical support to enter tests ordered by physicians, identify incomplete health maintenance measures, and preload new patient information | 14-item validated survey including burnout and subscale of depersonalization | | 4 mo | | IV |
| Contratto, 2017[10]^b,d^ | US | Quasi-experimental (single group pre-post intervention) mixed-methods | 7 | | Academic general internal medicine practice | | **Teamwork/Transitions:** Modifying workflow to include clerical support personnel for physician order entry | Burnout: MBI; Satisfaction | | 4 mo | | IV |
| Danila, 2018[57]^d^ | US | Pre-post intervention survey | 6 | | Physicians (3 rheumatologists and 3 endocrinologists)  Rheumatology and endocrinology clinics | | **Teamwork/Transitions:** Modified workflow to include scribes for documentation | JSS | | 6 wks | | IV |
| DeChant, 2019[11]^b,d,e^ | Multinational | Systematic review | NA | | NA | | **Teamwork/Transitions:** Varied | Varied | | Varied | | IV |
| Dunn, 2007[15]^b,d^ | US | Noncontrolled prospective intervention study | 22-32 | | Physicians  Primary care group | | **Teamwork/Transitions:** Data guided interventions and systematic improvement processes that included 1. leadership valuing physician well-being equal to quality of care and financial stewardship 2. physicians identifying factors that influenced well-being, followed by plans for improvement with accountability 3. measuring the well-being of physicians regularly using validated instruments. Managed MA training site to decrease turnover and quality of support for documentation | ACP/ASIM survey on physician satisfaction; MBI; QWC | | 6 yr | | IV |
| Dyrbye, 2016[13]^b^ | US | RCT | 290 | | Internal medicine and surgery specialists  Departments in two states | | **Teamwork:** Organization designated tasks and provided business time to cultivate well-being in job satisfaction, teamwork, relationships, personal strengths, problem solving, and positive emotions | MBI; Work Engagement Scale; Empowerment at Work Scale; Physician JSS | | 3 mo | | IB |
| Gidwani, 2017[19]^b,d^ | US | RCT | 4 | | Physicians  Academic family medicine clinic | | **Teamwork/Transitions:** Modified workflow to include use of scribes to draft all relevant documentation | Physician satisfaction, measured by a 5-item instrument that included physicians’ perceptions of chart quality and chart accuracy | | 1 yr | | IB |
| Goyal, 2018[21]^b,d,e^ | US | Prospective cohort | 7 | | General internal medicine physicians (attendings, supervising residents, first-year residents)  Major urban academic health center | | **Teamwork/Transitions:** Provided training and implementation of nurse-physician alphanumeric paging system to enhance team communication and interactions, and decrease frequency of disruptions | NR | | 2 w | | IIB |
| Heaton, 2016[23]^b^ | Multinational | Systematic review | NA | | NA | | **Teamwork:** Use of scribes for documentation | Provider satisfaction | | NA | | IIa |
| Hung, 2018[58]^d^ | US | Pre-post intervention survey | 680 | | Physicians  46 primary care departments in a large  ambulatory care delivery system | | **Teamwork/Transitions:** Lean-based workflow redesigns, which included co-locating physician and medical assistant dyads for documentation support, delegating significant responsibilities to nonphysician staff including MAs, and mandating greater coordination and communication among all care team members | MBI | | 3 yr | | IV |
| Imdieke, 2017[24]^b,d^ | US | Quasi-experimental, nonrandomized  pre- and post-intervention study | 2 | | Internal medicine physicians  Hospital-based, outpatient primary care clinic | | **Teamwork/Transitions:** Incorporated medical scribes in ambulatory clinic workflows to support provider documentation in the EHR | Provider satisfaction | | 4-6 wk | | IV |
| Keefer, 2018[29]^b,d^ | US | Pre-post intervention survey | NP | | Physicians  Pediatric hospital | | **Teamwork/Transitions:** Lean methodology to reduce overwork and burden for residents by modifying workflow including resequencing and standardizing. Additional full-time physicians were hired and schedule modifications were provided to reduce patient-to-attending ratio and added in-house evening and overnight resident supervision | MindTools | | 3 yr | | IV |
| Koshy, 2010[59]^d^ | US | Nonrandomized, static-group  comparison study | 5 | | Urologists, residents  Urology clinic within a single academic medical center | | **Teamwork/Transitions:** Modified workflows to use scribes to record electronic medical information throughout the patient–physician encounter | Physician acceptance and  satisfaction | | 10 mo | | IV |
| Linzer, 2015[60]^d^ | US | Cluster RCT | 166 (135 completed the study) | | Primary care physicians (family and general internists)  34 clinics in upper midwest and NYC | | **Teamwork/Transitions:** Projects to improve communication, changes in workflow to related to HIT documentation with MAs, and targeted quality improvement projects | Survey tools from MEMO and PWS | | 12 mo, 18 mo | | IIB |
| Linzer, 2016[61]^d^ | US | Cluster RCT | 165 | | Primary care physicians (family and general internists)  34 clinics in upper midwest and NYC | | **Teamwork/Transitions:** Quality improvements projects to improve communication between providers, workflow design with MAs, and chronic disease management | OWL | | 6 mo, 12 mo | | IIB |
| McCormick, 2018[37]^b,d^ | US | Pre-post intervention survey | 6 | | Urologists  Academic urology clinic | | **Teamwork/Transitions:** Modified workflows to use of scribes for documentation | Work satisfaction | | 3 mo | | IV |
| Mehta, 2018[38]^b,d,e^ | US | Pre-post intervention survey | NP | | Hospitalists  3 academic medicine sites | | **Teamwork/Transitions:** Use of scribes, modified EHR-related workflows, and customized EHR templates to reduce documentation | Custom satisfaction survey | | 6 mo | | IV |
| Mishra, 2018[42]^b,d^ | US | Dual-balanced crossover prospective cohort study | 18 | | Primary care providers without experience with scribes  Two medical centers in an integrated network | | **Teamwork/Transitions:** Assignment of medical scribes for electronic documentation (alternating 3 mo intervals with and without scribe support; randomized to start with or without support) and determine if documentation time is reduced for physicians; modified EHR-related workflow | 5-point Likert Scale | | 12 mo | | IIB |
| Pierce, 2017[62]^d^ | US | Pre-post intervention survey | 55 | | Physicians and advanced practice clinicians  Academic hospital | | **Teamwork/Transitions:** 13 team-based and organizational tactics to improve resilience, including expansion of leadership roles, faculty coaching for new hires, and value-based clinical schedule redesign to modify workflows. | NR | | 3 yr | | IV |
| Pozdnyakova, 2018[63]^d,e^ | US | Prospective, pre-post-pilot study | 6 | | General internal medicine faculty | | **Teamwork/Transitions:** Modified workflows to use of scribes for documentation using clinical task management system | Workplace satisfaction; burnout | | 1 wk | | IV |
| Quenot, 2012[64] | France | Longitudinal, monocentric, before-and-after, interventional study | 4 | | Physicians  ICU | | **Teamwork:** Intensive communication strategy regarding end of life practices in the ICU to alleviate stress for caregivers | MBI | | Post intervention | | IV |
| Shaw, 2017[65]^d^ | US | Pre-post intervention survey | NR | | Medical doctors  NR | | **Teamwork/Transitions:** Team-based primary care redesign, “Primary Care 2.0” (PC 2.0), with the goal of addressing the Quadruple Aim of healthcare (i.e., the Triple Aim plus reducing workforce burnout) with following components: 1. An expanded “Care Coordinator” (CC) role for medical assistants including scribing, population health management, and between-visit care management; 2. Health coaching and Motivational Interviewing; 3. “Lean” quality improvement to support a Learning Health System; 4. Telehealth; 5. Protected provider time for care coordination; and 6. An onsite extended interdisciplinary care team (i.e., mental health, pharmacy, physical therapy) | NR | | 5 mo | | IV |
| Shultz, 2015[66] | Multinational | Systematic review | NA | | Emergency department, urology, or cardiology clinicians | | **Teamwork:** Use of scribes | Clinician satisfaction | | NA | | IV |
| Was, 2016[67] | US | Pre-post intervention survey | 23 | | Residents  Large academic center | | **Teamwork:** Common space for residents (i.e., Gas Lounge) | NR | | Post intervention | | IV |
| West, 2014[68] | US | RCT | 74 | | Physicians  Department of medicine at the Mayo clinic | | **Teamwork:** 19 biweekly facilitated physician discussion groups incorporating elements of mindfulness, reflection, shared experience, and small group learning | JSS, empowerment at work scale; medical outcomes study short form health survey; MBI; perceived stress scale; scale of physician empathy | | 1 yr | | IB |
| Willard-Grace, 2017[55]^b,d^ | US | Cross-sectional | 236 | | Clinicians  County-run primary care clinics | | **Teamwork/Transitions:** A defined model of team-based care (enhanced roles for medical assistants, registered nurses, and behavioral health providers) including workflow redesign | MBI | | NR | | IV |
| ***Transitions* (N=31)** | | | | | | | | | | | | |
| Albadry, 2014[69] | Egypt | Cross-sectional | | 140 | Residents and assistant lecturers  Outpatient clinic | **Transitions:** *Six Sigma* methodology as quality improvement intervention | | | MBI | | 6 mo | IV |
| Amis, 2018[2]^b^ | UK | Pre-post intervention survey | | 13 | First year residents | **Transitions:** A checklist aimed to reduce the number of inappropriate prescribing tasks | | | Job satisfaction | | 3 weekends | IV |
| Baccei, 2020[5]^b,c^ | US | Pre-post intervention survey | | 6 | Musculoskeletal radiologists  Academic medical center hospital and outpatient location | **Teamwork/Transitions:** Lean methodology as part of quality improvement initiative in radiology department that included work schedule modifications for team-based care and modified workflow processes to decrease turnaround time of results | | | Custom satisfaction survey | | 6 mo | IV |
| Callahan, 2018[70] | US | Pre-post intervention survey | | 9 | Fellows  NR | **Transitions:** Bundle of evidence based  interventions to improve burnout and professional satisfaction, that were designed to fit the fellowship program | | | ESS, quality of life | | 6 mo | IV |
| Chapman, 2017[56]^c^ | US | Cross-sectional | | 886 | Primary care practices | **Teamwork/Transitions:** Employing medical assistants (MA) in an innovative model of care with workflow modifications for documentation | | | Satisfaction interview | | 4 yr | IV |
| Contratto, 2016[9]^b,c^ | US | Pre-post intervention survey | | 9 | Physicians  Urban academic general internal medicine primary care practice | **Teamwork/Transitions:** To evaluate the impact of modifying workflow to include full-time clerical support to enter tests ordered by physicians, identify incomplete health maintenance measures, and preload new patient information | | | 14-item validated survey including burnout and subscale of depersonalization | | 4 mo | IV |
| Contratto, 2017[10]^b,c^ | US | Quasi-experimental (single group pre-post intervention) mixed-methods | | 7 | Academic general internal medicine practice | **Teamwork/Transitions:** Modifying workflow to include clerical support personnel for physician order entry | | | Burnout: MBI; Satisfaction | | 4 mo | IV |
| Danila, 2018[57]^c^ | US | Pre-post intervention survey | | 6 | Physicians (3 rheumatologists and 3 endocrinologists)  Rheumatology and endocrinology clinics | **Teamwork/Transitions:** Modified workflow to include scribes for documentation | | | JSS | | 6 wks | IV |
| DeChant, 2019[11]^b,c,e^ | Multinational | Systematic review | | NA | NA | **Teamwork/Transitions:** Varied | | | Varied | | Varied | IV |
| Dunn, 2007[15]^b,c^ | US | Noncontrolled prospective intervention study | | 22-32 | Physicians  Primary care group | **Teamwork/Transitions:** Data guided interventions and systematic improvement processes that included 1. leadership valuing physician well-being equal to quality of care and financial stewardship 2. physicians identifying factors that influenced well-being, followed by plans for improvement with accountability 3. measuring the well-being of physicians regularly using validated instruments. Managed MA training site to decrease turnover and quality of support for documentation | | | ACP/ASIM survey on physician satisfaction; MBI; QWC | | 6 yr | IV |
| Giannini, 2013[71] | Italy | Pre-post intervention survey | | 71 | Doctors  ICU | **Transitions:** Increase in daily visiting time to at least 8 hours (policy change) | | | MBI; STAI | | 6 mo, 12 mo | IV |
| Gidwani, 2017[19]^b,c^ | US | RCT | | 4 | Physicians  Academic family medicine clinic | **Teamwork/Transitions:** Modified workflow to include use of scribes to draft all relevant documentation | | | Physician satisfaction, measured by a 5-item instrument that included physicians’ perceptions of chart quality and chart accuracy | | 1 yr | IB |
| Goyal, 2018[21]^b,c,e^ | US | Prospective cohort | | 7 | General internal medicine physicians (attendings, supervising residents, first-year residents)  Major urban academic health center | **Teamwork/Transitions:** Provided training and implementation of nurse-physician alphanumeric paging system to enhance team communication and interactions, and decrease frequency of disruptions | | | NR | | 2 w | IIB |
| Hung, 2018[58]^c^ | US | Pre-post intervention survey | | 680 | Physicians  46 primary care departments in a large  ambulatory care delivery system | **Teamwork/Transitions:** Lean-based workflow redesigns, which included co-locating physician and medical assistant dyads for documentation support, delegating significant responsibilities to nonphysician staff including MAs, and mandating greater coordination and communication among all care team members | | | MBI | | 3 yr | IV |
| Imdieke, 2017[24]^b,c^ | US | Quasi-experimental, nonrandomized  pre- and post-intervention study | | 2 | Internal medicine physicians  Hospital-based, outpatient primary care clinic | **Teamwork/Transitions:** Incorporated medical scribes in ambulatory clinic workflows to support provider documentation in the EHR | | | Provider satisfaction | | 4-6 wk | IV |
| Joseph, 2017[27]^b,e^ | US | Pre-post intervention survey | | NR | Providers  NR | **Transitions:** Process improvement aimed at improving individual clinician’s efficiency in using EHR by examining documentation time | | | NR | | NR | IV |
| Keefer, 2018[29]^b,c^ | US | Pre-post intervention survey | | NP | Physicians  Pediatric hospital | **Teamwork/Transitions:** Lean methodology to reduce overwork and burden for residents by modifying workflow including resequencing and standardizing. Additional full-time physicians were hired and schedule modifications were provided to reduce patient-to-attending ratio and added in-house evening and overnight resident supervision | | | MindTools | | 3 yr | IV |
| Koshy, 2010[59]^c^ | US | Nonrandomized, static-group  comparison study | | 5 | Urologists, residents  Urology clinic within a single academic medical center | **Teamwork/Transitions:** Modified workflows to use scribes to record electronic medical information throughout the patient–physician encounter | | | Physician acceptance and  satisfaction | | 10 mo | IV |
| Lee, 2017[72] | US | Pre-post intervention survey | | Baseline, 18  Postintervention, 15 | Neuroradiology fellows and neuroradiologists  Academic neuroradiology practice - part of a larger healthcare system with 6 hospitals and 80 outpatient imaging sites | **Transitions:** Image interpretive and non-image interpretive reading room workflows | | | 14-question survey, Likert scale rating 1-5 | | 1 mo | IV |
| Linzer, 2015[60]^c^ | US | Cluster RCT | | 166 (135 completed the study) | Primary care physicians (family and general internists)  34 clinics in upper midwest and NYC | **Teamwork/Transitions:** Projects to improve communication, changes in workflow to related to HIT documentation with MAs, and targeted quality improvement projects | | | Survey tools from MEMO and PWS | | 12 mo, 18 mo | IIB |
| Linzer, 2016[61]^c^ | US | Cluster RCT | | 165 | Primary care physicians (family and general internists)  34 clinics in upper midwest and NYC | **Teamwork/Transitions:** Quality improvements projects to improve communication between providers, workflow design with MAs, and chronic disease management | | | OWL | | 6 mo, 12 mo | IIB |
| Mazur, 2019[36]^b,e^ | US | Prospective cohort | | 38 | Internal medicine, family medicine, pediatrics, surgery, and other specialty residents and fellows  Large academic institution | **Transitions:** Quality improvement process to improve EHR with enhanced interface usability that automatically sorted all previously identified critical test results for patients. Also displayed info regarding patient status and decision support. Determined impact on time for task completion and examined workflow change using decision support | | | NASA-Task Load Index (Perceived workload and satisfaction) | | Post-intervention | IIB |
| McCormick, 2018[37]^b,c^ | US | Pre-post intervention survey | | 6 | Urologists  Academic urology clinic | **Teamwork/Transitions:** Modified workflows to use of scribes for documentation | | | Work satisfaction | | 3 mo | IV |
| Mehta, 2018[38]^b,c,e^ | US | Pre-post intervention survey | | NP | Hospitalists  3 academic medicine sites | **Teamwork/Transitions:** Use of scribes, modified EHR-related workflows, and customized EHR templates to reduce documentation | | | Custom satisfaction survey | | 6 mo | IV |
| Mishra, 2018[42]^b,c^ | US | Dual-balanced crossover prospective cohort study | | 18 | Primary care providers without experience with scribes  Two medical centers in an integrated network | **Teamwork/Transitions:** Assignment of medical scribes for electronic documentation (alternating 3 mo intervals with and without scribe support; randomized to start with or without support) and determine if documentation time is reduced for physicians; modified EHR-related workflow | | | 5-point Likert Scale | | 12 mo | IIB |
| Pierce, 2017[62]^c^ | US | Pre-post intervention survey | | 55 | Physicians and advanced practice clinicians  Academic hospital | **Teamwork/Transitions:** 13 team-based and organizational tactics to improve resilience, including expansion of leadership roles, faculty coaching for new hires, and value-based clinical schedule redesign to modify workflows. | | | NR | | 3 yr | IV |
| Pozdnyakova, 2018[63]^c,e^ | US | Prospective, pre-post-pilot study | | 6 | General internal medicine faculty | **Teamwork/Transitions:** Modified workflows to use of scribes for documentation using clinical task management system | | | Workplace satisfaction; burnout | | 1 wk | IV |
| Safavi, 2009[73] | Canada | Pre-post intervention survey | | 14 | Radiation oncologists (residents and staff)  Academic medical center | **Transitions:** Organization introduced a pilot of competence by design reporting by integrating entrustable professional activity assessments into their clinical workflow | | | NP | | End of block and clinical assessment period | IV |
| Shaw, 2017[65]^c^ | US | Pre-post intervention survey | | NR | Medical doctors  NR | **Teamwork/Transitions:** Team-based primary care redesign, “Primary Care 2.0” (PC 2.0), with the goal of addressing the Quadruple Aim of healthcare (i.e., the Triple Aim plus reducing workforce burnout) with following components: 1. An expanded “Care Coordinator” (CC) role for medical assistants including scribing, population health management, and between-visit care management; 2. Health coaching and Motivational Interviewing; 3. “Lean” quality improvement to support a Learning Health System; 4. Telehealth; 5. Protected provider time for care coordination; and 6. An onsite extended interdisciplinary care team (i.e., mental health, pharmacy, physical therapy) | | | NR | | 5 mo | IV |
| Willard-Grace, 2017[55]^b,c^ | US | Cross-sectional | | 236 | Clinicians  County-run primary care clinics | **Teamwork/Transitions:** A defined model of team-based care (enhanced roles for medical assistants, registered nurses, and behavioral health providers) including workflow redesign | | | MBI | | NR | IV |
| ***Technology* (N=17)** | | | | | | | | | | | | |
| Agha, 2010[74] | US | NR | | 9 | Pulmonary, rheumatology, and endocrine physicians  NR | **Technology:** EHR system implementation | | | NR | | NR | IV |
| Babbott, 2013[75] | US | Prospective | | 422 | Internal medicine and family medicine physicians | **Technology:** EHR system implementation and examination of its features | | | NR | | NR | IV |
| Beam, 2017[76] | US | Pre-post intervention survey | | 158 | Physicians  Neonatal intensive care unit (NICU) | **Technology:** Computerized provider order entry (CPOE) implementation | | | Job satisfaction | | 1 yr | IV |
| DeChant, 2019[11]^b,c,d^ | Multinational | Systematic review | | NA | NA | **Technology:** Varied | | | Varied | | Varied | IV |
| Ehrlich, 2016[16]^b^ | US | Pre-post intervention survey | | 25 | Ophthalmologists  Large academic ophthalmology department | **Technology:** EHR system implementation | | | 30 question survey using Likert scale rating; Job satisfaction | | 24 mo | IV |
| Goyal, 2018[21]^b,c,d^ | US | Prospective cohort | | 7 | General internal medicine physicians (attendings, supervising residents, first-year residents)  Major urban academic health center | **Technology:** Provided training and implementation of alphanumeric paging system | | | NR | | 2 w | IIB |
| Heyworth, 2012[77] | US | Pre-post intervention survey | | 163 | Primary care and specialty  NR | **Technology:** To measure predictors of physician satisfaction following EHR adoption | | | Massachusetts eHealth Collaborative Survey | | Post- intervention | IV |
| Joseph, 2017[27]^b,d^ | US | Pre-post intervention survey | | NR | Providers  NR | **Technology:** The impact of a brief, intensive technology adoption with modified workflows and training intervention aimed at improving individual clinician’s efficiency in using EHR | | | NR | | NR | IV |
| Lapointe, 2018[33] | US | Pre-post intervention survey | | 25 | Internal medicine residents  591-bed urban hospital | **Technology:** Improving communication using EHR-based text paging system with a modified workflow | | | Stress | | 6 mo | IV |
| Mazur, 2019[36]^b,d^ | US | Prospective cohort | | 38 | Internal medicine, family medicine, pediatrics, surgery, and other specialty residents and fellows  Large academic institution | **Technology:** Improve EHR with enhanced interface usability that automatically sorted all previously identified critical test results for patients. Also displayed info regarding patient status and decision support | | | NASA-Task Load Index (Perceived workload and satisfaction) | | Post-intervention | IIB |
| Mehta, 2018[38]^b,c,d^ | US | Pre-post intervention survey | | NP | Hospitalists  3 academic medicine sites | **Technology:** Customized EHR templates to reduce documentation | | | Custom satisfaction survey | | 6 mo | IV |
| Menachemi, 2009[78] | US | Cross-sectional | | 4,203 | Primary care physicians and clinical specialists  Outpatient settings | **Technology:** To evaluate the relationship between physician IT adoption and practice satisfaction | | | Survey using Likert scale questions on job/practice satisfaction | | NA | IV |
| Michelotti, 2013[79] | US | Pre-post intervention survey | | 59 | Faculty ophthalmologists  Large academic hospital | **Technology:** EHR system implementation | | | Custom satisfaction survey | | 3 mo | IV |
| Milenkiewicz, 2017[39]^b^ | US | Pre-post intervention survey | | NR | Physicians  Department of Addiction Medicine at Kaiser Permanente | **Technology:** To test the usability of an EHR tool to improve and standardize the documentation process | | | NR | | Post- intervention | IV |
| Payne, 2018[80] | US | RCT | | 31 | Internal medicine residents and attending hospitalists  Two academic medical centers | **Technology:** Use of Voice Generated Enhanced Electronic Note System (VGEENS) speech to text system for creating inpatient progress notes that are incorporated into the EHR | | | Modified Canada Health Infoway System and Use Assessment Survey (satisfaction) | | 9 mo | IIB |
| Pozdnyakova, 2018[63]^c,d^ | US | Prospective, pre-post-pilot study | | 6 | General internal medicine faculty | **Technology:** Modified workflows to use of scribes for documentation using clinical task management system | | | Workplace satisfaction; burnout | | 1 wk | IV |
| Wylie, 2014[81] | US | Cross-sectional | | 2365 | Primary care providers  Practice with more than 10 physicians | **Technology:** EHR adoption and implementation | | | Likert type scale questions regarding how EHR affected medical practice | | NA | IV |

Adapted from DeChant et al. (2019) [11]

Footnotes: Levels of evidence^a^: IB = Individual randomized controlled trial (RCT) (with narrow confidence interval); IIB = Individual cohort study (including low RCT; e.g., < 80% follow-up); IIA – Systematic review with homogeneity; IV = Case-series, poor-quality cohort, case-control studies, and systematic review with heterogeneity.[82]

^b^also categorized as time

^c^also categorized as teamwork

^d^also categorized as transitions

^e^also categorized as technology

Abbreviations: ACGME: American College of General Medical Education; ACP/ASIM: American College of Physicians/American Society of Internal Medicine; CC: Care coordinator; CI: Confidence interval; DSSQ: Dundee Stress State Questionnaire; EHR: Electronic health records; ESS: Epworth Sleepiness Scale; JoP: Joy of Practice; JSS: Physician Job Satisfaction Scale; ICU: Intensive care unit; MAs, medical assistants; MBI: Maslach-Jackson Burnout Inventory; MEMO study: Minimizing Error, Maximizing Outcome study; NR: Not reported; NYC: New York City; QoL: Quality of life; OR: Odds ratio; OWL: Office and Worklife Measures; PDA: Personal digital assistants; PSS, Physician Satisfaction Scale; PWS: Physician Work Satisfaction score; QWC: Quality Work Competency Survey; RCT: Randomized controlled trial; STAI: State-Trait Anxiety Inventory; UK: United Kingdom; US: United States; WTR: Working time restrictions.

Table 15. Subgroup analysis (n=38) study characteristics by 4Ts (time, teamwork, transitions, and technology) intervention type

| **Reference** | **Country** | **Study design** | **N** | **Population and setting** | **Type of intervention** | **Outcome and Measure Instrument** | **Follow-up** | **Level of evidence^a^** |
| --- | --- | --- | --- | --- | --- | --- | --- | --- |
| ***Time* (N=20)** | | | | | | | | |
| Amis, 2018[2]^d^ | UK | Pre-post intervention survey | 13 | First year residents | **Time:** A checklist aimed to reduce the number of inappropriate prescribing tasks | Job satisfaction | 3 weekends | IV |
| Baccei, 2020[5]^c,d^ | US | Pre-post intervention survey | 6 | Musculoskeletal radiologists  Academic medical center hospital and outpatient location | **Time:** Lean methodology as part of quality improvement initiative in radiology department that included work schedule modifications and processes to decrease turnaround time of results | Custom satisfaction survey | 6 mo | IV |
| Contratto, 2016[9]^c,d^ | US | Pre-post intervention survey | 9 | Primary care physicians  Urban academic general internal medicine primary care practice | **Time:** Evaluated documentation effort following a workflow change including full-time clerical support to enter tests ordered by physicians, identify incomplete health maintenance measures, and preload new patient information | 14-item validated survey including burnout and subscale of depersonalization | 4 mo | IV |
| Contratto, 2017[10]^c,d^ | US | Quasi-experimental (single group pre-post intervention) mixed-methods | 7 | Internal medicine physicians  Academic general internal medicine practice | **Time:** Modified workflow to include clerical support personnel for physician order entry and examined productivity | Burnout: MBI; Satisfaction | 4 mo | IV |
| DeChant, 2019[11]^c,d,e^ | Multinational | Systematic review | NA | NA | **Time:** Varied | Varied | Varied | IV |
| Dunn, 2007[15]^c,d^ | US | Noncontrolled prospective intervention study | 22-32 | Physicians  Primary care group | **Time:** Data guided interventions and systematic improvement processes that included providing schedule flexibility | ACP/ASIM survey on physician satisfaction; MBI; QWC | 6 yr | IV |
| Ehrlich, 2016[16]^e^ | US | Pre-post intervention survey | 25 | Ophthalmologists  Large academic ophthalmology department | **Time:** Examined documentation time, efficiency, and workflow after EHR system implementation | 30 question survey using Likert scale rating; Job satisfaction | 24 mo | IV |
| Gidwani, 2017[19]^c,d^ | US | RCT | 4 | Physicians  Academic family medicine clinic | **Time:** Scribes used to draft all relevant documentation and examined charting time and efficiency | Physician satisfaction, measured by a 5-item instrument that included physicians’ perceptions of chart quality and chart accuracy | 1 yr | IB |
| Goyal, 2018[21]^c,d,e^ | US | Prospective cohort | 7 | General internal medicine physicians (attendings, supervising residents, first-year residents)  Major urban academic health center | **Time:** Provided training and implementation of nurse-physician alphanumeric paging system to enhance communication and decrease frequency of disruptions | NR | 2 w | IIB |
| Heaton, 2016[23]^c^ | Multinational | Systematic review | NA | NA | **Time:** Scribes used for documentation and examined throughput in workflow | Provider satisfaction | NA | IIa |
| Imdieke, 2017[24]^c,d^ | US | Quasi-experimental, nonrandomized  pre- and post-intervention study | 2 | Internal medicine physicians  Hospital-based, outpatient primary care clinic | **Time:** Medical scribes used in an ambulatory clinic to support provider documentation in the EHR and examined documentation time | Provider satisfaction | 4-6 wk | IV |
| Joseph, 2017[27]^d,e^ | US | Pre-post intervention survey | NR | Providers  NR | **Time:** The impact of a brief, intensive technology adoption and training intervention aimed at improving individual clinician’s efficiency in using EHR by examining documentation time | NR | NR | IV |
| Keefer, 2018[29]^c,d^ | US | Pre-post intervention survey | NP | Physicians  Pediatric hospital | **Time:** Lean methodology to reduce overwork and burden for residents. Additional full-time physicians were hired and schedule modifications were provided to reduce patient-to-attending ratio and added in-house evening and overnight resident supervision | MindTools | 3 yr | IV |
| Lapointe, 2018[33]^d,e^ | US | Pre-post intervention survey | 25 | Internal medicine residents  591-bed urban hospital | **Time:** Improved communication using EHR-based text paging system and measured disruptions in time | Stress | 6 mo | IV |
| Mazur, 2019[36]^e^ | US | Prospective cohort | 38 | Internal medicine, family medicine, pediatrics, surgery, and other specialty residents and fellows  Large academic institution | **Time:** Quality improvement process to improve EHR with enhanced interface usability that automatically sorted all previously identified critical test results for patients. Also displayed info regarding patient status and decision support. Determined impact on time for task completion and examined workflow change using decision support | NASA-Task Load Index (Perceived workload and satisfaction) | Post-intervention | IIB |
| McCormick, 2018[37]^c,d^ | US | Pre-post intervention survey | 6 | Urologists  Academic urology clinic | **Time:** Scribes used for documentation and productivity | Work satisfaction | 3 mo | IV |
| Mehta, 2018[38]^c,d,e^ | US | Pre-post intervention survey | NP | Hospitalists  3 academic medicine sites | **Time:** Use of scribes, modified EHR-related workflows, and customized EHR templates to reduce documentation time, increase time spent for patient encounters, and improve work-life balance | Custom satisfaction survey | 6 mo | IV |
| Milenkiewicz, 2017[39]^e^ | US | Pre-post intervention survey | NR | Physicians  Department of Addiction Medicine at Kaiser Permanente | **Time:** Examined usability of an EHR tool to improve and standardize the documentation process including documentation time | NR | Post- intervention | IV |
| Mishra, 2018[42]^c,d^ | US | Dual-balanced crossover prospective cohort study | 18 | Primary care providers without experience with scribes  Two medical centers in an integrated network | **Time:** Assignment of medical scribes for electronic documentation (alternating 3 mo intervals with and without scribe support; randomized to start with or without support) and determine if documentation time is reduced for physicians | 5-point Likert Scale | 12 mo | IIB |
| Willard-Grace, 2017[55]^c,d^ | US | Cross-sectional | 236 | Clinicians  County-run primary care clinics | **Time:** A defined model of team-based care (enhanced roles for medical assistants, registered nurses, and behavioral health providers) is defined, workflow changed, and schedules redesigned | MBI | NR | IV |
| ***Teamwork* (N=23)** | | | | | | | | |
| Baccei, 2020[5]^b^ | US | Pre-post intervention survey | 6 | Musculoskeletal radiologists  Academic medical center hospital and outpatient location | **Teamwork/Transitions:** Lean methodology as part of quality improvement initiative in radiology department that included work schedule modifications for team-based care and modified workflow processes to decrease turnaround time of results | Custom satisfaction survey | 6 mo | IV |
| Chapman, 2017[56]^b,d^ | US | Cross-sectional | 886 | Primary care practices | **Teamwork/Transitions:** Employing medical assistants (MA) in an innovative model of care with workflow modifications for documentation | Satisfaction interview | 4 yr | IV |
| Contratto, 2016[9]^b,d^ | US | Pre-post intervention survey | 9 | Physicians  Urban academic general internal medicine primary care practice | **Teamwork/Transitions:** To evaluate the impact of modifying workflow to include full-time clerical support to enter tests ordered by physicians, identify incomplete health maintenance measures, and preload new patient information | 14-item validated survey including burnout and subscale of depersonalization | 4 mo | IV |
| Contratto, 2017[10] | US | Quasi-experimental (single group pre-post intervention) mixed-methods | 7 | Academic general internal medicine practice | **Teamwork/Transitions:** Modifying workflow to include clerical support personnel for physician order entry | Burnout: MBI; Satisfaction | 4 mo | IV |
| Danila, 2018[57]^d^ | US | Pre-post intervention survey | 6 | Physicians (3 rheumatologists and 3 endocrinologists)  Rheumatology and endocrinology clinics | **Teamwork/Transitions:** Modified workflow to include scribes for documentation | JSS | 6 wks | IV |
| DeChant, 2019[11]^b,d,e^ | Multinational | Systematic review | NA | NA | **Teamwork/Transitions:** Varied | Varied | Varied | IV |
| Dunn, 2007[15]^b,d^ | US | Noncontrolled prospective intervention study | 22-32 | Physicians  Primary care group | **Teamwork/Transitions:** Data guided interventions and systematic improvement processes that included 1. leadership valuing physician well-being equal to quality of care and financial stewardship 2. physicians identifying factors that influenced well-being, followed by plans for improvement with accountability 3. measuring the well-being of physicians regularly using validated instruments. Managed MA training site to decrease turnover and quality of support for documentation | ACP/ASIM survey on physician satisfaction; MBI; QWC | 6 yr | IV |
| Gidwani, 2017[19]^b,d^ | US | RCT | 4 | Physicians  Academic family medicine clinic | **Teamwork/Transitions:** Modified workflow to include use of scribes to draft all relevant documentation | Physician satisfaction, measured by a 5-item instrument that included physicians’ perceptions of chart quality and chart accuracy | 1 yr | IB |
| Goyal, 2018[21]^b,d,e^ | US | Prospective cohort | 7 | General internal medicine physicians (attendings, supervising residents, first-year residents)  Major urban academic health center | **Teamwork/Transitions:** Provided training and implementation of nurse-physician alphanumeric paging system to enhance team communication and interactions, and decrease frequency of disruptions | NR | 2 w | IIB |
| Heaton, 2016[23]^b^ | Multinational | Systematic review | NA | NA | **Teamwork:** Use of scribes for documentation | Provider satisfaction | NA | IIa |
| Hung, 2018[58]^d^ | US | Pre-post intervention survey | 680 | Physicians  46 primary care departments in a large  ambulatory care delivery system | **Teamwork/Transitions:** Lean-based workflow redesigns, which included co-locating physician and medical assistant dyads for documentation support, delegating significant responsibilities to nonphysician staff including MAs, and mandating greater coordination and communication among all care team members | MBI | 3 yr | IV |
| Imdieke, 2017[24]^b,d^ | US | Quasi-experimental, nonrandomized  pre- and post-intervention study | 2 | Internal medicine physicians  Hospital-based, outpatient primary care clinic | **Teamwork/Transitions:** Incorporated medical scribes in ambulatory clinic workflows to support provider documentation in the EHR | Provider satisfaction | 4-6 wk | IV |
| Keefer, 2018[29]^b,d^ | US | Pre-post intervention survey | NP | Physicians  Pediatric hospital | **Teamwork/Transitions:** Lean methodology to reduce overwork and burden for residents by modifying workflow including resequencing and standardizing. Additional full-time physicians were hired and schedule modifications were provided to reduce patient-to-attending ratio and added in-house evening and overnight resident supervision | MindTools | 3 yr | IV |
| Koshy, 2010[59]^d^ | US | Nonrandomized, static-group  comparison study | 5 | Urologists, residents  Urology clinic within a single academic medical center | **Teamwork/Transitions:** Modified workflows to use scribes to record electronic medical information throughout the patient–physician encounter | Physician acceptance and  satisfaction | 10 mo | IV |
| Linzer, 2015[60]^d^ | US | Cluster RCT | 166 (135 completed the study) | Primary care physicians (family and general internists)  34 clinics in upper midwest and NYC | **Teamwork/Transitions:** Projects to improve communication, changes in workflow to related to HIT documentation with MAs, and targeted quality improvement projects | Survey tools from MEMO and PWS | 12 mo, 18 mo | IIB |
| Linzer, 2016[61]^d^ | US | Cluster RCT | 165 | Primary care physicians (family and general internists)  34 clinics in upper midwest and NYC | **Teamwork/Transitions:** Quality improvements projects to improve communication between providers, workflow design with MAs, and chronic disease management | OWL | 6 mo, 12 mo | IIB |
| McCormick, 2018[37]^b,d^ | US | Pre-post intervention survey | 6 | Urologists  Academic urology clinic | **Teamwork/Transitions:** Modified workflows to use of scribes for documentation | Work satisfaction | 3 mo | IV |
| Mehta, 2018[38]^b,d,e^ | US | Pre-post intervention survey | NP | Hospitalists  3 academic medicine sites | **Teamwork/Transitions:** Use of scribes, modified EHR-related workflows, and customized EHR templates to reduce documentation | Custom satisfaction survey | 6 mo | IV |
| Mishra, 2018[42]^b,d^ | US | Dual-balanced crossover prospective cohort study | 18 | Primary care providers without experience with scribes  Two medical centers in an integrated network | **Teamwork/Transitions:** Assignment of medical scribes for electronic documentation (alternating 3 mo intervals with and without scribe support; randomized to start with or without support) and determine if documentation time is reduced for physicians; modified EHR-related workflow | 5-point Likert Scale | 12 mo | IIB |
| Pierce, 2017[62]^d^ | US | Pre-post intervention survey | 55 | Physicians and advanced practice clinicians  Academic hospital | **Teamwork/Transitions:** 13 team-based and organizational tactics to improve resilience, including expansion of leadership roles, faculty coaching for new hires, and value-based clinical schedule redesign to modify workflows. | NR | 3 yr | IV |
| Pozdnyakova, 2018[63]^d,e^ | US | Prospective, pre-post-pilot study | 6 | General internal medicine faculty | **Teamwork/Transitions:** Modified workflows to use of scribes for documentation using clinical task management system | Workplace satisfaction; burnout | 1 wk | IV |
| Shaw, 2017[65]^d^ | US | Pre-post intervention survey | NR | Medical doctors  NR | **Teamwork/Transitions:** Team-based primary care redesign, “Primary Care 2.0” (PC 2.0), with the goal of addressing the Quadruple Aim of healthcare (i.e., the Triple Aim plus reducing workforce burnout) with following components: 1. An expanded “Care Coordinator” (CC) role for medical assistants including scribing, population health management, and between-visit care management; 2. Health coaching and Motivational Interviewing; 3. “Lean” quality improvement to support a Learning Health System; 4. Telehealth; 5. Protected provider time for care coordination; and 6. An onsite extended interdisciplinary care team (i.e., mental health, pharmacy, physical therapy) | NR | 5 mo | IV |
| Willard-Grace, 2017[55]^b,d^ | US | Cross-sectional | 236 | Clinicians  County-run primary care clinics | **Teamwork/Transitions:** A defined model of team-based care (enhanced roles for medical assistants, registered nurses, and behavioral health providers) including workflow redesign | MBI | NR | IV |
| ***Transitions* (N=27)** | | | | | | | | |
| Amis, 2018[2]^b^ | UK | Pre-post intervention survey | 13 | First year residents | **Transitions:** Workflows changed to reduce the number of inappropriate prescribing tasks | Job satisfaction | 3 weekends | IV |
| Baccei, 2020[5]^b,c^ | US | Pre-post intervention survey | 6 | Musculoskeletal radiologists  Academic medical center hospital and outpatient location | **Teamwork/Transitions:** Lean methodology as part of quality improvement initiative in radiology department that included work schedule modifications for team-based care and modified workflow processes to decrease turnaround time of results | Custom satisfaction survey | 6 mo | IV |
| Chapman, 2017[56]^c^ | US | Cross-sectional | 886 | Primary care practices | **Teamwork/Transitions:** Employing medical assistants (MA) in an innovative model of care with workflow modifications for documentation | Satisfaction interview | 4 yr | IV |
| Contratto, 2016[9]^b,c^ | US | Pre-post intervention survey | 9 | Physicians  Urban academic general internal medicine primary care practice | **Teamwork/Transitions:** To evaluate the impact of modifying workflow to include full-time clerical support to enter tests ordered by physicians, identify incomplete health maintenance measures, and preload new patient information | 14-item validated survey including burnout and subscale of depersonalization | 4 mo | IV |
| Contratto, 2017[10]^b,c^ | US | Quasi-experimental (single group pre-post intervention) mixed-methods | 7 | Academic general internal medicine practice | **Teamwork/Transitions:** Modifying workflow to include clerical support personnel for physician order entry | Burnout: MBI; Satisfaction | 4 mo | IV |
| DeChant, 2019[11]^b,c,e^ | Multinational | Systematic review | NA | NA | **Teamwork/Transitions:** Varied | Varied | Varied | IIA |
| Dunn, 2007[15]^b,c^ | US | Noncontrolled prospective intervention study | 22-32 | Physicians  Primary care group | **Teamwork/Transitions:** Data guided interventions and systematic improvement processes that included 1. leadership valuing physician well-being equal to quality of care and financial stewardship 2. physicians identifying factors that influenced well-being, followed by plans for improvement with accountability 3. measuring the well-being of physicians regularly using validated instruments. Managed MA training site to decrease turnover and quality of support for documentation | ACP/ASIM survey on physician satisfaction; MBI; QWC | 6 yr | IV |
| Gidwani, 2017[19]^b,c^ | US | RCT | 4 | Physicians  Academic family medicine clinic | **Teamwork/Transitions:** Modified workflow to include use of scribes to draft all relevant documentation | Physician satisfaction, measured by a 5-item instrument that included physicians’ perceptions of chart quality and chart accuracy | 1 yr | IB |
| Goyal, 2018[21]^b,c,e^ | US | Prospective cohort | 7 | General internal medicine physicians (attendings, supervising residents, first-year residents)  Major urban academic health center | **Teamwork/Transitions:** Provided training and implementation of nurse-physician alphanumeric paging system to enhance team communication and interactions, and decrease frequency of disruptions | NR | 2 w | IIB |
| Hung, 2018[58]^c^ | US | Pre-post intervention survey | 680 | Physicians  46 primary care departments in a large  ambulatory care delivery system | **Teamwork/Transitions:** Lean-based workflow redesigns, which included co-locating physician and medical assistant dyads for documentation support, delegating significant responsibilities to nonphysician staff including MAs, and mandating greater coordination and communication among all care team members | MBI | 3 yr | IV |
| Imdieke, 2017[24]^b,c^ | US | Quasi-experimental, nonrandomized  pre- and post-intervention study | 2 | Internal medicine physicians  Hospital-based, outpatient primary care clinic | **Teamwork/Transitions:** Incorporated medical scribes in ambulatory clinic workflows to support provider documentation in the EHR | Provider satisfaction | 4-6 wk |  |
| Joseph, 2017[27]^b,e^ | US | Pre-post intervention survey | NR | Providers  NR | **Transitions:** Process improvement aimed at improving individual clinician’s efficiency in using EHR by examining documentation time | NR | NR | IV |
| Keefer, 2018[29]^b,c^ | US | Pre-post intervention survey | NP | Physicians  Pediatric hospital | **Teamwork/Transitions:** Lean methodology to reduce overwork and burden for residents by modifying workflow including resequencing and standardizing. Additional full-time physicians were hired and schedule modifications were provided to reduce patient-to-attending ratio and added in-house evening and overnight resident supervision | MindTools | 3 yr | IV |
| Koshy, 2010[59]^c^ | US | Nonrandomized, static-group  comparison study | 5 | Urologists, residents  Urology clinic within a single academic medical center | **Teamwork/Transitions:** Modified workflows to use scribes to record electronic medical information throughout the patient–physician encounter | Physician acceptance and  satisfaction | 10 mo | IV |
| Lapointe, 2018[33]^b,e^ | US | Pre-post intervention survey | 25 | Internal medicine residents  591-bed urban hospital | **Transitions:** Improving communication using EHR-based text paging system with a modified workflow | Stress | 6 mo | IV |
| Lee, 2017[72] | US | Pre-post intervention survey | Baseline, 18  Postintervention, 15 | Neuroradiology fellows and neuroradiologists  Academic neuroradiology practice - part of a larger healthcare system with 6 hospitals and 80 outpatient imaging sites | **Transitions:** Image interpretive and non-image interpretive reading room workflows including EHR-related tasks | 14-question survey, Likert scale rating 1-5 | 1 mo | IV |
| Linzer, 2015[60]^c^ | US | Cluster RCT | 166 (135 completed the study) | Primary care physicians (family and general internists)  34 clinics in upper midwest and NYC | **Teamwork/Transitions:** Projects to improve communication, changes in workflow to related to HIT documentation, and targeted quality improvement projects | Survey tools from MEMO and PWS | 12 mo, 18 mo | IIB |
| Linzer, 2016[61]^c^ | US | Cluster RCT | 165 | Primary care physicians (family and general internists)  34 clinics in upper midwest and NYC | **Teamwork/Transitions:** Quality improvements projects to improve communication between providers, workflow design, and chronic disease management | OWL | 6 mo, 12 mo | IIB |
| Mazur, 2019[36]^b,e^ | US | Prospective cohort | 38 | Internal medicine, family medicine, pediatrics, surgery, and other specialty residents and fellows  Large academic institution | **Transitions:** Quality improvement process to improve EHR with enhanced interface usability that automatically sorted all previously identified critical test results for patients. Also displayed info regarding patient status and decision support. Determined impact on time for task completion and examined workflow change using decision support | NASA-Task Load Index (Perceived workload and satisfaction) | Post-intervention | IIB |
| McCormick, 2018[37]^b,c^ | US | Pre-post intervention survey | 6 | Urologists  Academic urology clinic | **Teamwork/Transitions:** Modified workflows to use of scribes for documentation | Work satisfaction | 3 mo | IV |
| Mehta, 2018[38]^b,c,e^ | US | Pre-post intervention survey | NP | Hospitalists  3 academic medicine sites | **Teamwork/Transitions:** Use of scribes, modified EHR-related workflows, and customized EHR templates to reduce documentation | Custom satisfaction survey | 6 mo | IV |
| Mishra, 2018[42]^b,c^ | US | Dual-balanced crossover prospective cohort study | 18 | Primary care providers without experience with scribes  Two medical centers in an integrated network | **Teamwork/Transitions:** Assignment of medical scribes for electronic documentation (alternating 3 mo intervals with and without scribe support; randomized to start with or without support) and determine if documentation time is reduced for physicians; modified EHR-related workflow | 5-point Likert Scale | 12 mo | IIB |
| Pierce, 2017[62]^c^ | US | Pre-post intervention survey | 55 | Physicians and advanced practice clinicians  Academic hospital | **Teamwork/Transitions:** 13 team-based and organizational tactics to improve resilience, including expansion of leadership roles, faculty coaching for new hires, and value-based clinical schedule redesign to modify workflows. | NR | 3 yr | IV |
| Pozdnyakova, 2018[63]^c,e^ | US | Prospective, pre-post-pilot study | 6 | General internal medicine faculty | **Teamwork/Transitions:** Modified workflows to use of scribes for documentation using clinical task management system | Workplace satisfaction; burnout | 1 wk | IV |
| Shaw, 2017[65]^c^ | US | Pre-post intervention survey | NR | Medical doctors  NR | **Teamwork/Transitions:** Team-based primary care redesign, “Primary Care 2.0” (PC 2.0), with the goal of addressing the Quadruple Aim of healthcare (i.e., the Triple Aim plus reducing workforce burnout) with following components: 1. An expanded “Care Coordinator” (CC) role for medical assistants including scribing, population health management, and between-visit care management; 2. Health coaching and Motivational Interviewing; 3. “Lean” quality improvement to support a Learning Health System; 4. Telehealth; 5. Protected provider time for care coordination; and 6. An onsite extended interdisciplinary care team (i.e., mental health, pharmacy, physical therapy) | NR | 5 mo | IV |
| Willard-Grace, 2017[55]^b,c^ | US | Cross-sectional | 236 | Clinicians  County-run primary care clinics | **Teamwork/Transitions:** A defined model of team-based care (enhanced roles for medical assistants, registered nurses, and behavioral health providers) including workflow redesign | MBI | NR | IV |
| ***Technology* (N=17)** | | | | | | | | |
| Agha, 2010[74] | US | NR | 9 | Pulmonary, rheumatology, and endocrine physicians  NR | **Technology:** EHR system implementation | NR | NR | IV |
| Babbott, 2013[75] | US | Prospective | 422 | Internal medicine and family medicine physicians | **Technology:** EHR system implementation and examination of its features | NR | NR | IV |
| Beam, 2017[76] | US | Pre-post intervention survey | 158 | Physicians  Neonatal intensive care unit (NICU) | **Technology:** Computerized provider order entry (CPOE) implementation | Job satisfaction | 1 yr | IV |
| DeChant, 2019[11]^b,c,d^ | Multinational | Systematic review | NA | NA | **Technology:** Varied | Varied | Varied | IV |
| Ehrlich, 2016[16]^b^ | US | Pre-post intervention survey | 25 | Ophthalmologists  Large academic ophthalmology department | **Technology:** EHR system implementation | 30 question survey using Likert scale rating; Job satisfaction | 24 mo | IV |
| Goyal, 2018[21]^b,c,d^ | US | Prospective cohort | 7 | General internal medicine physicians (attendings, supervising residents, first-year residents)  Major urban academic health center | **Technology:** Provided training and implementation of alphanumeric paging system | NR | 2 w | IIB |
| Heyworth, 2012[77] | US | Pre-post intervention survey | 163 | Primary care and specialty  NR | **Technology:** To measure predictors of physician satisfaction following EHR adoption | Massachusetts eHealth Collaborative Survey | Post- intervention | IV |
| Joseph, 2017[27]^b,d^ | US | Pre-post intervention survey | NR | Providers  NR | **Technology:** The impact of a brief, intensive technology adoption with modified workflows and training intervention aimed at improving individual clinician’s efficiency in using EHR | NR | NR | IV |
| Lapointe, 2018[33]^d^ | US | Pre-post intervention survey | 25 | Internal medicine residents  591-bed urban hospital | **Technology:** Improving communication using EHR-based text paging system with a modified workflow | Stress | 6 mo | IV |
| Mazur, 2019[36]^b^ | US | Prospective cohort | 38 | Internal medicine, family medicine, pediatrics, surgery, and other specialty residents and fellows  Large academic institution | **Technology:** Improve EHR with enhanced interface usability that automatically sorted all previously identified critical test results for patients. Also displayed info regarding patient status and decision support | NASA-Task Load Index (Perceived workload and satisfaction) | Post-intervention | IIB |
| Mehta, 2018[38]^b,c,d^ | US | Pre-post intervention survey | NP | Hospitalists  3 academic medicine sites | **Technology:** Customized EHR templates to reduce documentation | Custom satisfaction survey | 6 mo | IV |
| Menachemi, 2009[78] | US | Cross-sectional | 4,203 | Primary care physicians and clinical specialists  Outpatient settings | **Technology:** To evaluate the relationship between physician IT adoption and practice satisfaction | Survey using Likert scale questions on job/practice satisfaction | NA | IV |
| Michelotti, 2013[79] | US | Pre-post intervention survey | 59 | Faculty ophthalmologists  Large academic hospital | **Technology:** EHR system implementation | Custom satisfaction survey | 3 mo | IV |
| Milenkiewicz, 2017[39]^b^ | US | Pre-post intervention survey | NR | Physicians  Department of Addiction Medicine at Kaiser Permanente | **Technology:** To test the usability of an EHR tool to improve and standardize the documentation process | NR | Post- intervention | IV |
| Payne, 2018[80] | US | RCT | 31 | Internal medicine residents and attending hospitalists  Two academic medical centers | **Technology:** Use of Voice Generated Enhanced Electronic Note System (VGEENS) speech to text system for creating inpatient progress notes that are incorporated into the EHR | Modified Canada Health Infoway System and Use Assessment Survey (satisfaction) | 9 mo | IIB |
| Pozdnyakova, 2018[63]^c,d^ | US | Prospective, pre-post-pilot study | 6 | General internal medicine faculty | **Technology:** Modified workflows to use of scribes for documentation using clinical task management system | Workplace satisfaction; burnout | 1 wk | IV |
| Wylie, 2014[81] | US | Cross-sectional | 2,365 | Primary care providers  Practice with more than 10 physicians | **Technology:** EHR adoption and implementation | Likert type scale questions regarding how EHR affected medical practice | NA | IV |

Table adapted from DeChant et al. (2019)[11]

Footnotes: Levels of evidence^a^: IB = Individual randomized controlled trial (RCT) (with narrow confidence interval); IIB = Individual cohort study (including low RCT; e.g., < 80% follow-up); IIA – Systematic review with homogeneity; IV = Case-series, poor-quality cohort, case-control studies, and systematic review with heterogeneity.[82]

^b^also categorized as time

^c^also categorized as teamwork

^d^also categorized as transitions

^e^also categorized as technology

Abbreviations: ACGME: American College of General Medical Education; ACP/ASIM: American College of Physicians/American Society of Internal Medicine; CC: Care coordinator; CI: Confidence interval; DSSQ: Dundee Stress State Questionnaire; EHR: Electronic health records; ESS: Epworth Sleepiness Scale; JoP: Joy of Practice; JSS: Physician Job Satisfaction Scale; ICU: Intensive care unit; MAs, medical assistants; MBI: Maslach-Jackson Burnout Inventory; MEMO study: Minimizing Error, Maximizing Outcome study; NR: Not reported; NYC: New York City; QoL: Quality of life; OR: Odds ratio; OWL: Office and Worklife Measures; PDA: Personal digital assistants; PSS, Physician Satisfaction Scale; PWS: Physician Work Satisfaction score; QWC: Quality Work Competency Survey; RCT: Randomized controlled trial; STAI: State-Trait Anxiety Inventory; UK: United Kingdom; US: United States; WTR: Working time restrictions.

**References**

1. Ali NA, Wolf KM, Hammersley J, et al. Continuity of care in intensive care units: a cluster-randomized trial of intensivist staffing. American Journal of Respiratory and Critical Care Medicine 2011;**184**(7):803-08

2. Amis SM, Osicki THE. Can patient safety be improved by reducing the volume of "inappropriate prescribing tasks" handed over to out-of-hours junior doctors? International Journal of General Medicine 2018;**11**:105-12 doi: 10.2147/IJGM.S153227[published Online First: Epub Date]|.

3. Amutio A, Martínez-Taboada C, Delgado LC, Hermosilla D, Mozaz MJ. Acceptability and Effectiveness of a Long-Term Educational Intervention to Reduce Physicians' Stress-Related Conditions. Journal of Continuing Education in the Health Professions 2015;**35**(4):255-60 doi: 10.1097/ceh.0000000000000002[published Online First: Epub Date]|.

4. Ares WJ, Maroon JC, Jankowitz BT. In Pursuit of Balance: The UPMC Neurosurgery Wellness Initiative. World Neurosurg 2019;**132**:e704-e09 doi: 10.1016/j.wneu.2019.08.034[published Online First: Epub Date]|.

5. Baccei SJ, Henderson SR, Lo HS, Reynolds K. Using Quality Improvement Methodology to Reduce Costs while Improving Efficiency and Provider Satisfaction in a Busy, Academic Musculoskeletal Radiology Division. J Med Syst 2020;**44**(6):104 doi: 10.1007/s10916-020-01569-8[published Online First: Epub Date]|.

6. Bragard I, Etienne AM, Merckaert I, Libert Y, Razavi D. Efficacy of a communication and stress management training on medical residents' self-efficacy, stress to communicate and burnout: a randomized controlled study. J Health Psychol 2010;**15**(7):1075-81 doi: 10.1177/1359105310361992[published Online First: Epub Date]|.

7. Butow P, Cockburn J, Girgis A, et al. Increasing oncologists' skills in eliciting and responding to emotional cues: evaluation of a communication skills training program. Psycho-Oncology 2008;**17**(3):209-18 doi: 10.1002/pon.1217[published Online First: Epub Date]|.

8. Butow P, Brown R, Aldridge J, et al. Can consultation skills training change doctors' behaviour to increase involvement of patients in making decisions about standard treatment and clinical trials: a randomized controlled trial. Health Expectations 2015;**18**(6):2570-83 doi: 10.1111/hex.12229[published Online First: Epub Date]|.

9. Contratto EC, Estrada C, Romp KG, et al. The impact of physician order entry clerical support on physician satisfaction and productivity. Journal of General Internal Medicine 2016;**31**(2 SUPPL. 1):S912-S13

10. Contratto E, Romp K, Estrada CA, Agne A, Willett LL. Physician Order Entry Clerical Support Improves Physician Satisfaction and Productivity. Southern Medical Journal 2017;**110**(5):363-68 doi: 10.14423/SMJ.0000000000000645[published Online First: Epub Date]|.

11. DeChant PF, Acs A, Rhee KB, et al. Effect of Organization-Directed Workplace Interventions on Physician Burnout: A Systematic Review. Mayo Clinic Proceedings: Innovations, Quality & Outcomes 2019;**3**(4):384-408 doi: 10.1016/j.mayocpiqo.2019.07.006[published Online First: Epub Date]|.

12. Desai SV, Asch DA, Bellini LM, et al. Education Outcomes in a Duty-Hour Flexibility Trial in Internal Medicine. New England Journal of Medicine 2018;**378**(16):1494-508 doi: 10.1056/NEJMoa1800965[published Online First: Epub Date]|.

13. Dyrbye LN, West CP, Richards ML, Ross HJ, Satele D, Shanafelt T. A randomized, controlled study of an online intervention to promote job satisfaction and well-being among physicians. Burnout Research 2016;**3**(3):69-75

14. Dyrbye LN, Shanafelt TD, Gill PR, Satele DV, West CP. Effect of a Professional Coaching Intervention on the Well-being and Distress of Physicians: A Pilot Randomized Clinical Trial. JAMA Intern Med 2019 doi: 10.1001/jamainternmed.2019.2425[published Online First: Epub Date]|.

15. Dunn PM, Arnetz BB, Christensen JF, Homer L. Meeting the imperative to improve physician well-being: assessment of an innovative program. Journal of General Internal Medicine 2007;**22**(11):1544-52

16. Ehrlich JR, Michelotti M, Blachley TS, et al. A Two-Year Longitudinal Assessment of Ophthalmologists' Perceptions after Implementing an Electronic Health Record System. Applied Clinical Informatics 2016;**7**(4):930-45 doi: 10.4338/ACI-2016-05-RA-0075[published Online First: Epub Date]|.

17. Fassiotto MA. A time banking system to support workplace flexibility. International Conference on Physician Health. Boston, MA, 2016:17.

18. Garland A, Roberts D, Graff L. Twenty-four–Hour Intensivist Presence: A Pilot Study of Effects on Intensive Care Unit Patients, Families, Doctors, and Nurses. American Journal of Respiratory and Critical Care Medicine 2012;**185**(7):738-43

19. Gidwani R, Nguyen C, Kofoed A, et al. Impact of Scribes on Physician Satisfaction, Patient Satisfaction, and Charting Efficiency: A Randomized Controlled Trial. Annals of Family Medicine 2017;**15**(5):427-33 doi: 10.1370/afm.2122[published Online First: Epub Date]|.

20. Goodman MJ, Schorling JB. A Mindfulness Course Decreases Burnout and Improves Well-Being among Healthcare Providers. The International Journal of Psychiatry in Medicine 2012;**43**(2):119-28 doi: 10.2190/PM.43.2.b[published Online First: Epub Date]|.

21. Goyal P, Cohen-Mekelburg S, Egan C, et al. New uses of old technology: Can nurse-pagers improve communication between resident-physicians and nurses. Appl Nurs Res 2018;**44**:1-5 doi: 10.1016/j.apnr.2018.08.003[published Online First: Epub Date]|.

22. Hart D, Paetow G, Zarzar R. Does Implementation of a Corporate Wellness Initiative Improve Burnout? West J Emerg Med 2019;**20**(1):138-44 doi: 10.5811/westjem.2018.10.39677[published Online First: Epub Date]|.

23. Heaton HA, Castaneda-Guarderas A, Trotter ER, Erwin PJ, Bellolio MF. Effect of scribes on patient throughput, revenue, and patient and provider satisfaction: a systematic review and meta-analysis. American Journal of Emergency Medicine 2016;**34**(10):2018-28 doi: 10.1016/j.ajem.2016.07.056[published Online First: Epub Date]|.

24. Imdieke BH, Martel ML. Integration of Medical Scribes in the Primary Care Setting: Improving Satisfaction. Journal of Ambulatory Care Management 2017;**40**(1):17-25 doi: 10.1097/JAC.0000000000000168[published Online First: Epub Date]|.

25. Ireland MJ, Clough B, Gill K, Langan F, O'Connor A, Spencer L. A randomized controlled trial of mindfulness to reduce stress and burnout among intern medical practitioners. Med Teach 2017;**39**(4):409-14 doi: 10.1080/0142159x.2017.1294749[published Online First: Epub Date]|.

26. Isaksson Ro KE, Tyssen R, Hoffart A, Sexton H, Aasland OG, Gude T. A three-year cohort study of the relationships between coping, job stress and burnout after a counselling intervention for help-seeking physicians. BMC Public Health 2010;**10**:213 doi: 10.1186/1471-2458-10-213[published Online First: Epub Date]|.

27. Joseph C. Rebooting the joy of practice = clinical/operational leadership + workflow standardization + technology. American Conference on Physician Health. San Francisco, CA, 2017:29-30.

28. Kakarala R, Smith SJ, Barreto E, Donelan K, Palamara K. When Coaching Meets Mentoring: Impact of Incorporating Coaching into an Existing Mentoring Program at a Community Hospital. Cureus 2018;**10**(8):e3138 doi: 10.7759/cureus.3138[published Online First: Epub Date]|.

29. Keefer L, Beck M, O'Hara C. Lean in: Our secrets to decreasing provider stress, maximizing efficiency on a pediatric hospitalist service. Journal of Hospital Medicine 2018;**13**(4)

30. Have Accreditation Council for Graduate Medical Education duty hour limits made a difference? A re-examination of resident sleep, mental health, education, and safety seven years later. Journal of Investigative Medicine; 2011. LIPPINCOTT WILLIAMS & WILKINS 530 WALNUT ST, PHILADELPHIA, PA 19106-3621 USA.

31. Krasner MS, Epstein RM, Beckman H, et al. Association of an educational program in mindful communication with burnout, empathy, and attitudes among primary care physicians. Jama 2009;**302**(12):1284-93 doi: 10.1001/jama.2009.1384[published Online First: Epub Date]|.

32. Landrigan CP, Fahrenkopf AM, Lewin D, et al. Effects of the accreditation council for graduate medical education duty hour limits on sleep, work hours, and safety. Pediatrics 2008;**122**(2):250-58

33. Lapointe R, Bhesania S, Tanner T, Peruri A, Mehta P. An Innovative Approach to Improve Communication and Reduce Physician Stress and Burnout in a University Affiliated Residency Program. Journal of Medical Systems 2018;**42**(7):117 doi: 10.1007/s10916-018-0956-z[published Online First: Epub Date]|.

34. Lucas BP, Trick WE, Evans AT, et al. Effects of 2-vs 4-week attending physician inpatient rotations on unplanned patient revisits, evaluations by trainees, and attending physician burnout: a randomized trial. JAMA 2012;**308**(21):2199-207

35. Martins AE, Davenport MC, Paz Del Valle M, et al. Impact of a brief intervention on the burnout levels of pediatric residents. J Pediatr (Rio J) 2011;**87**(6):493-98

36. Mazur LM, Mosaly PR, Moore C, Marks L. Association of the Usability of Electronic Health Records With Cognitive Workload and Performance Levels Among Physicians. JAMA network open 2019;**2**(4):e191709‐ doi: 10.1001/jamanetworkopen.2019.1709[published Online First: Epub Date]|.

37. McCormick BJ, Deal A, Borawski KM, et al. Implementation of medical scribes in an academic urology practice: an analysis of productivity, revenue, and satisfaction. World Journal of Urology 2018;**36**(10):1691-97 doi: 10.1007/s00345-018-2293-8[published Online First: Epub Date]|.

38. Mehta S, Johnston R, Yadav S, Cadotte R, Maranda M, Mogannam J. Scribes in hospital medicine-a powerful value-added resource! Journal of Hospital Medicine 2018;**13**(4)

39. Milenkiewicz RV. The less clicks the better: Improving the documentation workflow process for physicians. American Conference on Physician Health. San Francisco, CA, 2017:97-98.

40. Milstein JM, Raingruber BJ, Bennett SH, Kon AA, Winn CA, Paterniti DA. Burnout assessment in house officers: evaluation of an intervention to reduce stress. Med Teach 2009;**31**(4):338-41 doi: 10.1080/01421590802208552[published Online First: Epub Date]|.

41. Minichiello V, Hayer S, Gillespie B, Goss M, Barrett B. Developing a Mindfulness Skills-Based Training Program for Resident Physicians. Fam Med 2020;**52**(1):48-52 doi: 10.22454/FamMed.2020.461348[published Online First: Epub Date]|.

42. Mishra P, Kiang JC, Grant RW. Association of Medical Scribes in Primary Care With Physician Workflow and Patient Experience. JAMA internal medicine 2018;**178**(11):1467‐72 doi: 10.1001/jamainternmed.2018.3956[published Online First: Epub Date]|.

43. Moeller MF. Practice Refresh: A 1-month intervention to rebuild physician efficiency and wellness. American Conference on Physician Health. San Francisco, CA, 2017:27-28.

44. Moffatt-Bruce SD, Nguyen MC, Steinberg B, Holliday S, Klatt M. Interventions to Reduce Burnout and Improve Resilience: Impact on a Health System's Outcomes. Clin Obstet Gynecol 2019;**62**(3):432-43 doi: 10.1097/grf.0000000000000458[published Online First: Epub Date]|.

45. Morrow G, Burford B, Carter M, Illing J. Have restricted working hours reduced junior doctors' experience of fatigue? A focus group and telephone interview study. BMJ Open 2014;**4**(3) doi: 10.1136/bmjopen-2013-004222 FULL TEXT LINK [http://dx.doi.org/10.1136/bmjopen-2013-004222[published](http://dx.doi.org/10.1136/bmjopen-2013-004222%5bpublished) Online First: Epub Date]|.

46. Parshuram CS, Amaral ACKB, Ferguson ND, et al. Patient safety, resident well-being and continuity of care with different resident duty schedules in the intensive care unit: a randomized trial. Canadian Medical Association Journal 2015;**187**(5):321-29

47. Ripp JA, Bellini L, Fallar R, Bazari H, Katz JT, Korenstein D. The impact of duty hours restrictions on job burnout in internal medicine residents: a three-institution comparison study. Academic Medicine 2015;**90**(4):494-99

48. Runyan C, Savageau JA, Potts S, Weinreb L. Impact of a family medicine resident wellness curriculum: a feasibility study. Med Educ Online 2016;**21**:30648 doi: 10.3402/meo.v21.30648[published Online First: Epub Date]|.

49. Schuh LA, Khan MA, Harle H, et al. Pilot trial of IOM duty hour recommendations in neurology residency programs Unintended consequences. Neurology 2011;**77**(9):883-87

50. Shea JA, Bellini LM, Dinges DF, et al. Impact of protected sleep period for internal medicine interns on overnight call on depression, burnout, and empathy. Journal of Graduate Medical Education 2014;**6**(2):256-63

51. Sherlock C, John C. Adaptation Practice: Teaching doctors how to cope with stress, anxiety and depression by developing resilience. British Journal of Medical Practitioners 2016;**9**(2):a916

52. Sood A, Prasad K, Schroeder D, Varkey P. Stress management and resilience training among Department of Medicine faculty: a pilot randomized clinical trial. J Gen Intern Med 2011;**26**(8):858-61 doi: 10.1007/s11606-011-1640-x[published Online First: Epub Date]|.

53. Tucker P, Brown M, Dahlgren A, et al. The impact of junior doctors. Scandinavian Journal of Work, Environment & Health 2010:458-65

54. Verweij H, Waumans RC, Smeijers D, et al. Mindfulness-based stress reduction for GPs: results of a controlled mixed methods pilot study in Dutch primary care. Br J Gen Pract 2016;**66**(643):e99-105 doi: 10.3399/bjgp16X683497[published Online First: Epub Date]|.

55. Willard-Grace R. Clinician perception of team support, burnout and “doability” of primary care. American Conference on Physician Health. San Francisco, CA, 2017:20.

56. Chapman SA, Blash LK. New Roles for Medical Assistants in Innovative Primary Care Practices. Health Services Research 2017;**52 Suppl 1**:383-406 doi: 10.1111/1475-6773.12602[published Online First: Epub Date]|.

57. Danila MI, Melnick JA, Curtis JR, Menachemi N, Saag KG. Use of Scribes for Documentation Assistance in Rheumatology and Endocrinology Clinics: Impact on Clinic Workflow and Patient and Physician Satisfaction. Journal of Clinical Rheumatology 2018;**24**(3):116-21 doi: 10.1097/RHU.0000000000000620[published Online First: Epub Date]|.

58. Hung DY, Harrison MI, Truong Q, Du X. Experiences of primary care physicians and staff following lean workflow redesign. BMC Health Services Research 2018;**18**(1):274 doi: 10.1186/s12913-018-3062-5[published Online First: Epub Date]|.

59. Koshy S, Feustel PJ, Hong M, Kogan BA. Scribes in an ambulatory urology practice: patient and physician satisfaction. Journal of Urology 2010;**184**(1):258-62 doi: 10.1016/j.juro.2010.03.040[published Online First: Epub Date]|.

60. Linzer M, Poplau S, Grossman E, et al. A Cluster Randomized Trial of Interventions to Improve Work Conditions and Clinician Burnout in Primary Care: Results from the Healthy Work Place (HWP) Study. Journal of General Internal Medicine 2015;**30**(8):1105-11 doi: 10.1007/s11606-015-3235-4

10.1007/s11606-015-3235-4. Epub 2015 Feb 28.[published Online First: Epub Date]|.

61. Linzer M, Poplau S, Brown R, et al. Do Work Condition Interventions Affect Quality and Errors in Primary Care? Results from the Healthy Work Place Study. Journal of General Internal Medicine 2016;**32**(1):56-61 doi: 10.1007/s11606-016-3856-2

10.1007/s11606-016-3856-2. Epub 2016 Sep 9.[published Online First: Epub Date]|.

62. Pierce RG. A team-based and organizational framework for fostering resilience and well-being in academic hospital medicine. American Conference on Physician Health. San Francisco, CA, 2017:1-2.

63. Pozdnyakova A, Laiteerapong N, Volerman A, et al. Impact of Medical Scribes on Physician and Patient Satisfaction in Primary Care. Journal of General Internal Medicine 2018;**33**(7):1109-15 doi: 10.1007/s11606-018-4434-6[published Online First: Epub Date]|.

64. Quenot J-P, Rigaud J-P, Prin S, et al. Suffering among carers working in critical care can be reduced by an intensive communication strategy on end-of-life practices. Intensive Care Medicine 2012;**38**(1):55-61

65. Shaw JG, Brown-Johnson C, Chan G, Mahoney M, Winget M. Physician burnout and joy of practice: Early mixed methods findings from the implementation of stanford primary care 2.0. Journal of General Internal Medicine 2017;**32**(2 Supplement 1):S272

66. Shultz CG, Holmstrom HL. The use of medical scribes in health care settings: a systematic review and future directions. Journal of the American Board of Family Medicine 2015;**28**(3):371-81 doi: 10.3122/jabfm.2015.03.140224[published Online First: Epub Date]|.

67. Was A. The ideal gas lounge: Boosting resident happiness with empowerment and common space improvements. International Conference on Physician Health. Boston, MA, 2016:39.

68. West CP, Dyrbye LN, Rabatin JT, et al. Intervention to promote physician well-being, job satisfaction, and professionalism: a randomized clinical trial. JAMA Internal Medicine 2014;**174**(4):527-33

69. Albadry AA, Sleem AN, Montasser NA, Naggar E-SAE-. Effect of Quality Improvement Intervention on Occupational Burnout in Mansoura University Hospitals, Egypt. WELL-Med Conference. Alexandroupolis, Greece, 2014:43.

70. An innovative, evidence-based, fellow-driven bundle to improve professional satisfaction and wellness. American Thoracic Society 2018; San Diego, CA. American Journal of Respiratory and Critical Care Medicine.

71. Giannini A, Miccinesi G, Prandi E, Buzzoni C, Borreani C. Partial liberalization of visiting policies and ICU staff: a before-and-after study. Intensive Care Medicine 2013;**39**(12):2180-87

72. Lee MH, Schemmel AJ, Pooler BD, et al. Radiology Workflow Dynamics: How Workflow Patterns Impact Radiologist Perceptions of Workplace Satisfaction. Academic Radiology 2017;**24**(4):483-87 doi: 10.1016/j.acra.2016.08.027 FULL TEXT LINK [http://dx.doi.org/10.1016/j.acra.2016.08.027[published](http://dx.doi.org/10.1016/j.acra.2016.08.027%5bpublished) Online First: Epub Date]|.

73. Safavi A, Sienna J, Strang BK, Hann C. CHARACTERIZING THE INITIATION, COMPLETION, AND INTEGRATION OF ENTRUSTABLE PROFESSIONAL ACTIVITY ASSESSMENTS IN RADIATION ONCOLOGY RESIDENCY TRAINING AND CLINICAL WORKFLOW: AN INSTITUTIONAL PILOT OF COMPETENCE BY DESIGN. Radiotherapy and Oncology 2019;**139**:S85 doi: 10.1016/S0167-8140(19)33263-3[published Online First: Epub Date]|.

74. Agha Z, Roter D, Laud P, et al. Patient-centered communication and physicians use of electronic medical records. Journal of General Internal Medicine 2010;**25 SUPPL. 3**:S344 doi: 10.1007/s11606-010-1338-5 FULL TEXT LINK [http://dx.doi.org/10.1007/s11606-010-1338-5[published](http://dx.doi.org/10.1007/s11606-010-1338-5%5bpublished) Online First: Epub Date]|.

75. Babbott S, Baier L, Linzer M, et al. Electronic health records and physician stress in office based practice. JAMIA 2013:1-7

76. Beam KS, Cardoso M, Sweeney M, Binney G, Weingart SN. Examining Perceptions of Computerized Physician Order Entry in a Neonatal Intensive Care Unit. Applied Clinical Informatics 2017;**8**(2):337-47 doi: 10.4338/ACI-2016-09-RA-0153[published Online First: Epub Date]|.

77. Heyworth L, Zhang F, Jenter CA, et al. Physician satisfaction following electronic health record adoption in three Massachusetts communities. Interactive Journal of Medical Research 2012;**1**(2)

78. Menachemi N, Powers TL, Brooks RG. The role of information technology usage in physician practice satisfaction. Health Care Management Review 2009;**34**(4):364-71

79. Michelotti M, Weizer J, Blachley T, et al. Ophthalmologists' attitudes on implementing an electronic health record system. Investigative Ophthalmology and Visual Science 2013;**54**(15)

80. Payne TH, Alonso WD, Markiel JA, et al. Using voice to create inpatient progress notes: effects on note timeliness, quality, and physician satisfaction. JAMIA open 2018;**1**(2):218‐26 doi: 10.1093/jamiaopen/ooy036[published Online First: Epub Date]|.

81. Wylie MC, Baier RR, Gardner RL. Perceptions of electronic health record implementation: a statewide survey of physicians in Rhode Island. The American Journal of Medicine 2014;**127**(10):1010.e21-7 doi: 10.1016/j.amjmed.2014.06.011

10.1016/j.amjmed.2014.06.011. Epub 2014 Jun 16.[published Online First: Epub Date]|.

82. Oxford Centre for Evidence-based Medicine – Levels of Evidence (March 2009). Secondary Oxford Centre for Evidence-based Medicine – Levels of Evidence (March 2009) 2009. <http://www.cebm.net/blog/2009/06/11/oxford-centre-evidence-based-medicine-levels-evidence-march-2009/>.
